# Development and Validation of an IoF-Based Sensing Architecture for Microclimate Monitoring: Quantifying Spatial ET_0_ Bias in Mediterranean Viticulture

**DOI:** 10.3390/s26165154

**Published:** 2026-08-14

**Authors:** Okan Oral, Mustafa Cakir, Nuri Caglayan, Huseyin Kursat Celik

**Affiliations:** 1 Department of Mechanical Engineering, Faculty of Engineering, Akdeniz University, Antalya 07070, Türkiye; okan@akdeniz.edu.tr; 2 Department of UAV Technology, Iskenderun Technical University, Iskenderun 31200, Türkiye; mustafa.cakir@iste.edu.tr; 3 Department of Agricultural Machinery and Technology Engineering, Akdeniz University, Antalya 07070, Türkiye; hkcelik@akdeniz.edu.tr

**Keywords:** Internet of Farming (IoF), sensing architecture, microclimate monitoring, precision viticulture, ET_0_ bias correction, sensor benchmarking, spatial heterogeneity

## Abstract

This study presents and validates a modular Internet of Farming (IoF) sensing architecture designed for high-fidelity microclimate monitoring to address the spatial biases in reference evapotranspiration (ET_0_) estimation for precision viticulture. Deployed in a Mediterranean vineyard in Antalya, Türkiye, the system demonstrated high operational robustness with a 98.6% data completion rate over an annual cycle. Comparative analysis between field-level IoF data and regional meteorological station (TSMS) records revealed a systematic overestimation of ET_0_ by regional networks (MBE = −0.695 mm/day, MAPE = 10.56%). Sensitivity analysis identified discrepancies in wind speed (36.6%), net radiation (31%), and relative humidity (30%) as the primary physical drivers of this spatial bias, with air temperature serving as a seasonal index for their integrated intensification rather than as a direct dominant contributor (~2.4%). Results indicate that reliance on regional data would lead to an irrigation surplus of approximately 97.5 mm (11.4%) during the growing season. The findings underscore the critical role of localized sensing layers in resolving microclimatic heterogeneities that regional grids fail to capture within the specific site and year examined. While these results motivate further investigation into IoF-based architectures as a potentially transferable framework for ET_0_ bias correction, broader generalisation to other crops, climate regimes, and management systems remains a promising avenue for future multi-site, multi-year validation rather than an established conclusion of the present study.

## 1. Introduction

The depletion of freshwater resources and the increasing unpredictability of precipitation patterns pose critical challenges to sustainable agricultural production worldwide. Among the strategies to address water scarcity, precision irrigation management stands out as one of the most effective approaches, as it directly links water application to actual crop requirements rather than relying on generalized schedules [1,2]. Central to precision irrigation is the accurate estimation of reference evapotranspiration (ET_0_), which serves as the primary input for calculating crop water demand (ET_C_) through crop coefficient (K_C_) relationships [3]. Widely recognized as the benchmark for estimation, the FAO Penman–Monteith method [4] necessitates a comprehensive set of inputs, including air temperature, relative humidity, wind speed, and solar radiation [5].

In practice, these meteorological inputs are typically sourced from government-operated weather stations, which serve broad geographic areas. Specifically, in the context of Türkiye, the climatic parameters utilized in the study are traditionally analyzed based on official records obtained from the national meteorological network, managed by the Turkish State Meteorological Service (TSMS). However, considerable evidence suggests that spatial heterogeneity in microclimatic conditions, driven by differences in topography, land cover, proximity to urban heat islands, and local vegetation patterns, can introduce significant deviations between regional meteorological observations and actual field-level conditions [6].

Achieving accurate results in ET_C_ parameter optimization through various computational and statistical ET_0_ estimation methodologies [7,8,9,10,11,12,13,14,15] remains essential for the effective implementation of precision irrigation strategies. However, there remains a need for site-specific benchmark comparison of these models using localized, real-time data to overcome the inherent limitations of distant TSMS stations.

The integration of Internet of Farming (IoF) frameworks and low-cost sensor platforms has recently become instrumental in the high-fidelity acquisition of environmental variables that govern evapotranspiration dynamics. Technological advances have made it both technically and economically feasible to deploy field-level monitoring systems tailored to individual agricultural plots [16,17,18]. Several studies have demonstrated the potential of these architectures; for instance, Abba et al. [19] reported satisfactory performance for a smart IoT-based irrigation system, while Rodríguez-Robles et al. [20] developed an autonomous sensor network for rural environments. Similarly, Rajak et al. [21] emphasized the role of smart sensors in enhancing crop productivity. In a complementary approach, Ikram et al. [22] demonstrated IoT-based decision systems for yield maximization. Studies in the Thessaly plain [23] further integrated surface measurements with remote-sensing data to evaluate irrigation needs against the FAO framework. Furthermore, the deployment of Machine Learning (ML) algorithms alongside these IoT frameworks has shown superior efficacy in predictive modeling of ET values [24,25,26,27]. Such precision in forecasting serves as a cornerstone for optimizing irrigation scheduling and enhancing overall agricultural productivity [28]. Complementing these efforts, recent systematic reviews have synthesized trends and challenges in IoT-based irrigation sensing more broadly [29], while advances in computationally efficient ET estimation approaches continue to support real-time smart-agriculture applications [30].

While the broader literature predominantly employs the term Internet of Things (IoT) to describe sensor-based connectivity in agricultural contexts, the present study adopts the more domain-specific concept of IoF. IoF extends the generic IoT paradigm by explicitly embedding agricultural operational logic, including crop-specific data acquisition, agrometeorological processing, and irrigation decision-support, into the system architecture [17]. Unlike general-purpose IoT deployments, IoF frameworks are designed from the outset to capture, transmit, and interpret the biological and physical variables that govern plant-water relations under field conditions, making the term more precise and scientifically appropriate for the system described herein. This terminological distinction carries substantive scientific implications that directly shape the present study’s design and contribution. At the system design level, the IoF paradigm mandates the co-integration of crop-specific sensing priorities, agrometeorological processing logic, and irrigation decision-support within a unified architecture, commitments directly reflected in the sensor selection, canopy-level placement, and duty-cycle management strategy employed here; a generic IoT deployment, being agnostic to plant-water relations, would not by design require the simultaneous acquisition of microclimate, soil moisture, and irrigation water quality parameters. At the scientific contribution level, the IoF framing shifts the core research question from whether sensors can transmit data reliably (an IoT-level concern) to whether field-embedded agrometeorological sensing can resolve the spatial ET_0_ bias introduced by reliance on distant regional stations; this reframing positions the agronomic consequences of spatial data mismatch, rather than sensor engineering performance alone, as the primary scientific output of the study. To better position the proposed system within the current state of the art, representative low-cost agricultural sensing platforms reported in the recent literature are compared in Table 1.

As summarized in Table 1, previous low-cost agricultural IoT sensing platforms have primarily focused on individual functionalities such as microclimate monitoring, smart irrigation, greenhouse automation, or ET_0_ estimation. The novelty of the proposed system does not arise from the use of individual hardware components, which are mature and commercially available, but from their functional integration into an application-specific IoF framework for precision irrigation management. In contrast, the proposed IoF framework combines autonomous solar-powered operation, real-time vineyard microclimate monitoring, FAO-56 Penman–Monteith-based ET_0_ estimation, validation against TSMS, and irrigation-oriented decision support within a unified three-tier architecture, thereby providing a more comprehensive framework for precision irrigation management.

To further situate the proposed framework within domestic and international practice, recent reviews and field deployments illustrate the diversity of application-specific IoF/IoT sub-implementations that have emerged internationally, including wireless energy-balance sensor networks for crop ET determination [31], IoT-based smart reference-ET estimation combined with ensemble machine learning [32], IoT-based microclimate monitoring for irrigation-water optimization [33], IoT-driven fertigation scheduling and greenhouse microclimate monitoring [34], UAV- and ground-based canopy transpiration mapping integrated with weather data [35], and IoT-integrated digital-twin frameworks for controlled-environment microclimate control [36] and an electronic-atmometer-based instrumentation design for evapotranspiration measurement [37], alongside broader systematic reviews of IoT sensing trends in irrigation management [29] and in precision agriculture more generally [38]. Within the Turkish agricultural engineering literature, comparable IoT-based prototypes have similarly been reported [17], though generally without the crop-specific agrometeorological integration that defines the IoF paradigm adopted here. This diversity of deployed systems underscores that IoF, unlike generic IoT, is not defined by a single hardware configuration but by the functional co-integration of agrometeorological sensing and irrigation decision logic, precisely the criterion against which the present framework is positioned in Table 1.

Despite these advances, existing low-cost agricultural sensing platforms generally focus on individual functionalities such as environmental monitoring, irrigation automation, or ET_0_ estimation. Consequently, comprehensive IoF frameworks capable of integrating autonomous field operation, continuous microclimate monitoring, FAO-56 Penman–Monteith-based ET_0_ estimation, validation against regional meteorological observations, and irrigation-oriented decision support remain scarce, particularly under commercial vineyard conditions. Much of the current literature focuses on system design, technical validation, and algorithmic efficiency rather than quantifying the agronomic impact of localized versus regional data. Few studies have systematically quantified the magnitude and seasonal dynamics of ET_0_ deviations arising from spatial mismatch or translated these deviations into practical irrigation management implications. These questions are particularly vital for high-value crops like the grapevine (*Vitis vinifera* L.). Türkiye ranks among the top grape-producing countries globally, with a production of approximately 2.52 million tons in 2025 [39,40]. In the Mediterranean climate of the Antalya region, characterized by hot, dry summers, efficient water management is essential, as both over-irrigation and under-irrigation adversely affect yield and quality [41,42].

To address this discrepancy, the present study introduces an IoF-based weather station (Field) designed to capture microclimatic variables, providing a robust framework for the systematic benchmark comparison of localized ET_0_ and ETc estimations against official regional observations. To this end, the following research questions are addressed:

- What is the statistical magnitude and temporal pattern of ET_0_ deviations between TSMS-derived regional data and localized sources?
- Which meteorological variables function as the primary drivers of these discrepancies?
- What are the practical implications of these variances for crop water requirement estimation and irrigation scheduling?

To achieve this, the study focuses on the following four objectives:

- System development: Design and field deployment of a low-cost, modular IoF-based sensing architecture for real-time acquisition of climatic, soil, and water quality data in a commercial vineyard, providing the localized data infrastructure necessary for all subsequent analyses.
- Statistical quantification: Systematic comparison of ET_0_ estimates from localized IoF data versus regional TSMS data to answer: What is the statistical magnitude of ET_0_ deviations between TSMS-derived regional data and localized sources?
- Temporal pattern analysis: Regression-based identification of seasonal deviation dynamics and their meteorological drivers, with explicit attribution of the primary physical contributors to ET_0_ discrepancy, addressing: Which meteorological variables function as the primary drivers of these discrepancies ?
- Irrigation impact assessment: Translation of ET_0_ deviations into ET_C_ differences for grapevine, directly addressing: What are the practical implications of these variances for crop water requirement estimation and irrigation scheduling?

The proposed layered architecture, comprising sensing, processing, and decision-support layers—was developed for deployment in commercial vineyards. Although its transferability to other crops, climatic regions, and management systems appears promising, further validation through multi-site studies is required.

## 2. Materials and Methods

### 2.1. Study Area and Experimental Setup

Field data collection was conducted in a vineyard located in the Antalya province of Türkiye (36°53′ N, 30°38′ E, altitude 39 m) covering the full annual cycle from January to December 2021. The region experiences a Mediterranean climate, characterized by hot, dry summers and mild, wet winters. To evaluate the spatial mismatch in meteorological data, observations from the national meteorological network, managed by the Turkish State Meteorological Service (TSMS), were utilized as a regional reference. Specifically, the datasets were retrieved from the TSMS Antalya Regional Station (Station No: 17302), the nearest automated weather station located approximately 15 km away in Antalya city center. The comparative analysis in this study specifically utilizes synchronized daily datasets from both the localized IoF field station and the TSMS records for the identical 2021 calendar year. This framework serves to benchmark in situ IoF sensing capabilities against a standardised regional reference and highlights the profound impact of microclimatic variations on irrigation requirement modeling. This regional baseline provides quality-controlled datasets, including air temperature, relative humidity, wind speed, and solar radiation, standardized according to international meteorological protocols.

### 2.2. Monitoring System Architecture

**Figure 1:**
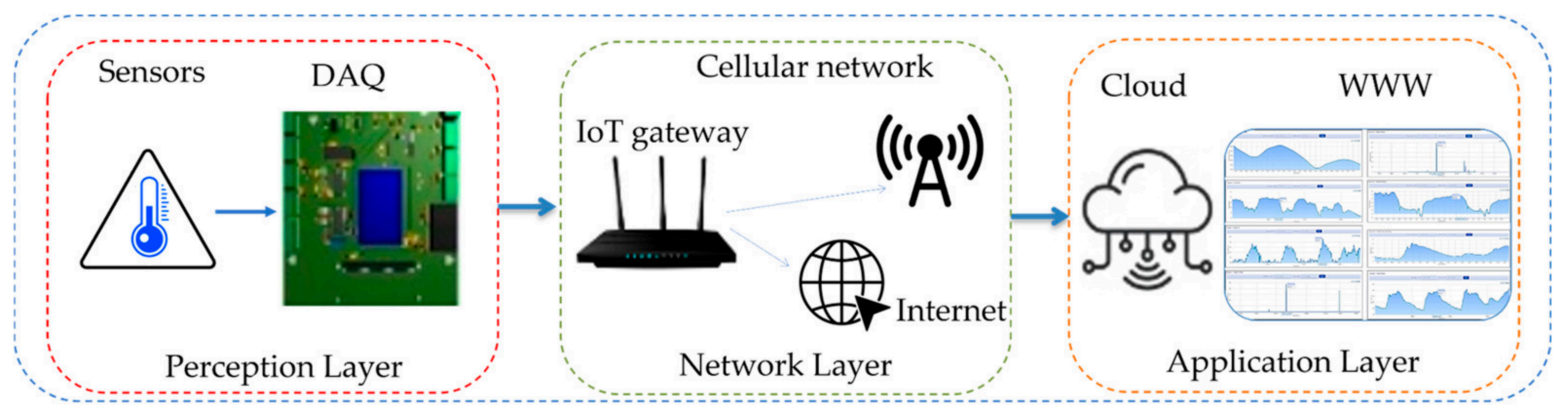
Overall architecture of the proposed Internet of Farming (IoF) platform based on the perception, network, and application layers. The perception layer acquires real-time environmental, soil, and irrigation-related measurements through distributed sensors connected to the data acquisition (DAQ) subsystem. The network layer manages data aggregation, wireless communication, and transmission through the GSM/GPRS cellular network using HTTP POST requests with JSON-formatted payloads. The application layer receives, stores, processes, and visualizes the transmitted data on the cloud server hosted at Akdeniz University, where measurements are archived in a MySQL relational database and made available to users through web- and mobile-based interfaces for irrigation monitoring and decision support.

Signal acquisition and electrical interfaces. As summarized in Table 2, the proposed IoF platform integrates heterogeneous sensing devices employing different electrical output characteristics according to the measured parameter. Sensors such as air temperature, relative humidity, soil temperature, soil moisture, electrical conductivity, pH, barometric pressure, and solar radiation generate analog or conditioned analog measurement signals that are digitized by the DAQ subsystem using a 12-bit analog-to-digital converter (ADC) with 4096 discrete quantization levels. In contrast, pulse-based sensors, including the rainfall gauge and Hall-effect wind speed sensor, are acquired through digital pulse counting, while wind direction measurements are obtained from the corresponding directional sensing circuitry. Following acquisition, all sensor measurements are synchronized by the ESP32 controller, assigned timestamps, and assembled into a unified dataset prior to cloud transmission. The system utilizes a three-tier IoF architecture (perception, network, and application), as illustrated in Figure 1.

#### 2.2.1. Perception Layer

**Figure 2:**
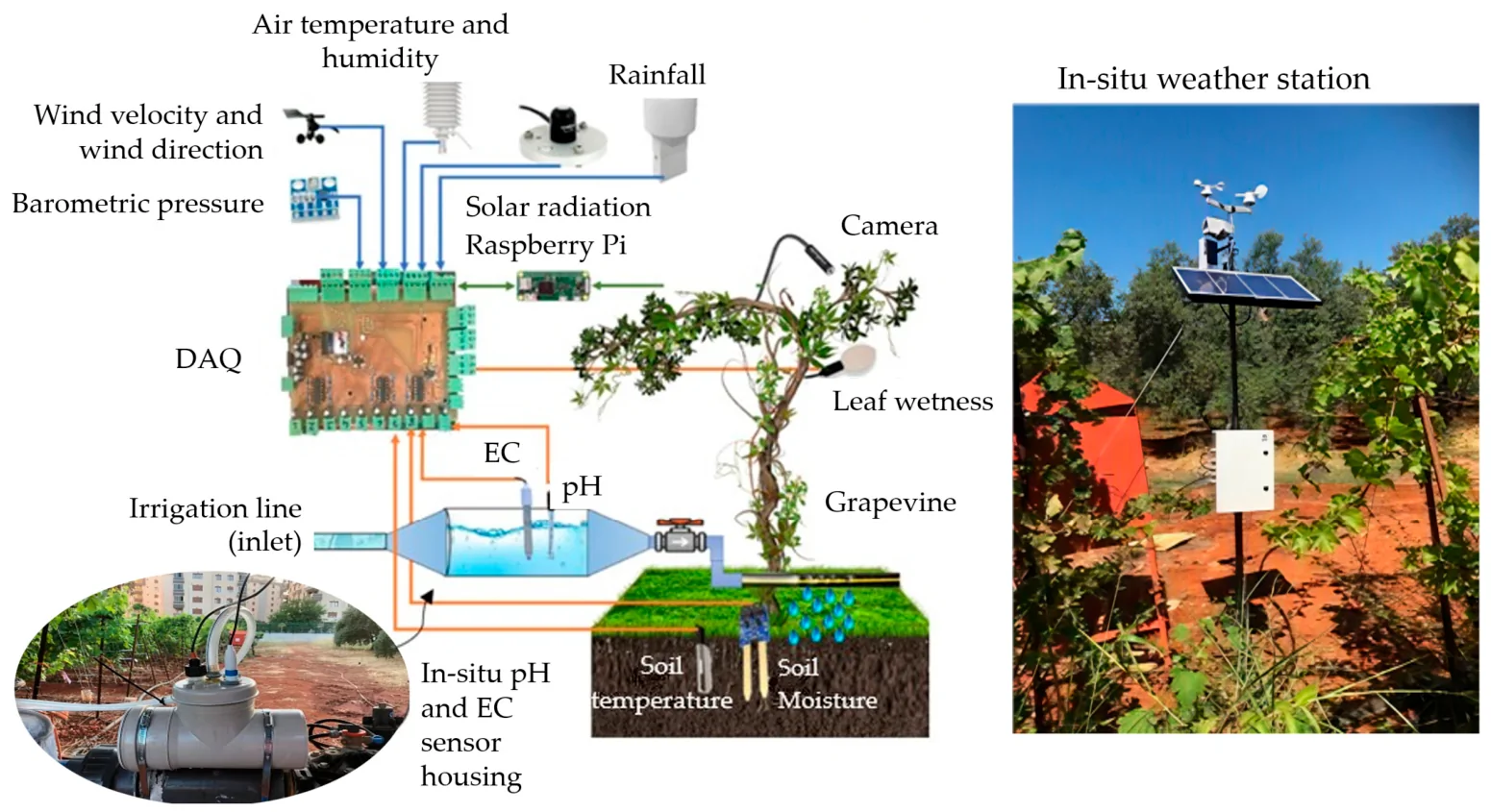
Functional block diagram of the IoF field station illustrating the integration of the sensing units, DAQ subsystem, ESP32 controller, Raspberry Pi Zero Wireless module, GSM/GPRS communication unit, camera module, and power management system. Analog sensor signals are digitized by the 12-bit DAQ subsystem, while digital sensors communicate directly with the controller through their native interfaces. The controller performs sensor polling, timestamp generation, preliminary data validation, and packet preparation prior to wireless transmission to the cloud platform.

This physical layer serves as the primary data collection unit, consisting of a modular multi-sensor field station and a Data Acquisition (DAQ) system. The field station integrates 12 sensors mounted on a telescopic mast extending up to 3.5 m, facilitating the real-time acquisition of microclimatic, edaphic, and hydrological variables directly from the vineyard (Figure 2). This layer is specifically responsible for capturing critical parameters such as air temperature, relative humidity, and solar radiation at the crop canopy level. Detailed technical specifications for the integrated sensors, including their respective measurement ranges and accuracy levels, are provided in Table 2.

The system’s hardware core is based on a hybrid architecture that integrates an ESP32-D0WD dual-core processor (80–240 MHz; Espressif Systems, Shanghai, China) with a Raspberry Pi Zero Wireless module (1 GHz; Raspberry Pi Foundation, Cambridge, UK), enabling a high-speed data transmission rate of 150 Mbps. For visual monitoring, a 24-bit pipe-type camera was incorporated to capture real-time images of leaf and fruit surfaces. The entire field station is autonomously powered by a 42 W photovoltaic panel coupled with a 12 V/7 Ah battery.

The ESP32 microcontroller collects and processes sensor measurements before transmitting the acquired data to the network layer. Signal acquisition and electrical interfaces: The proposed IoF platform integrates heterogeneous sensing devices with different electrical output characteristics and communication interfaces, as summarized in Table 2. Environmental parameters such as air temperature, relative humidity, and atmospheric pressure are measured simultaneously via a digital I^2^C communication interface. Dedicated soil temperature measurements are obtained using a digital temperature sensor based on the One-Wire communication protocol. Additionally, wind speed and rainfall are measured using digital sensors that provide pulse-based outputs, which are processed by the ESP32 controller through pulse-counting algorithms.

Analog-output sensors are acquired through the integrated data acquisition (DAQ) subsystem using the 12-bit analog-to-digital converter (ADC) of the ESP32 platform, providing 4096 discrete quantization levels. The capacitive soil moisture sensor generates an analog voltage output between 0 and 4.2 V, while solar radiation, pH, electrical conductivity, and leaf wetness sensors provide analog measurement signals that are converted into digital values before data processing. Following acquisition, all sensor measurements are synchronized by the ESP32 controller, assigned timestamps, and combined into a unified dataset for subsequent wireless transmission to the cloud platform.

#### 2.2.2. Network Layer

Acting as an IoF-driven bridge between the physical sensing layer and the digital cloud environment, this communication architecture facilitates the seamless transmission of digitized field measurements to the application server. Within the field station’s internal hardware layout, sub-modules and physical sensors interface with the main micro-controller via One-Wire and $I^{2}C$ digital communication protocols.

**Figure 3:**
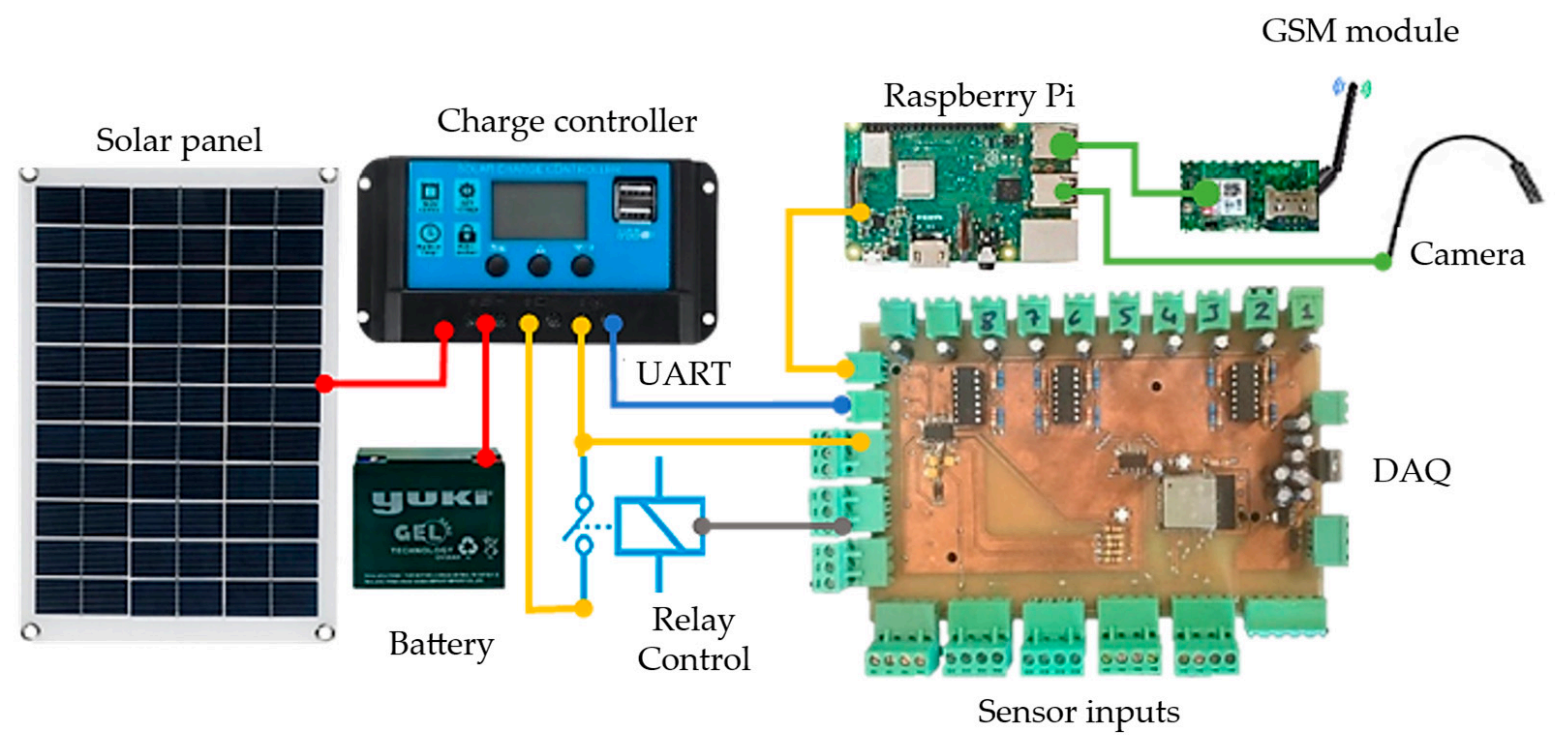
Circuit schematic of the proposed IoF field station showing the electrical interconnection among the sensors, ESP32 microcontroller, Raspberry Pi Zero Wireless module, GSM/GPRS communication module, data acquisition circuitry, power management unit, and peripheral devices. The diagram illustrates the complete hardware architecture responsible for sensor acquisition, signal conditioning, data processing, communication, and autonomous field operation.

**Figure 4:**
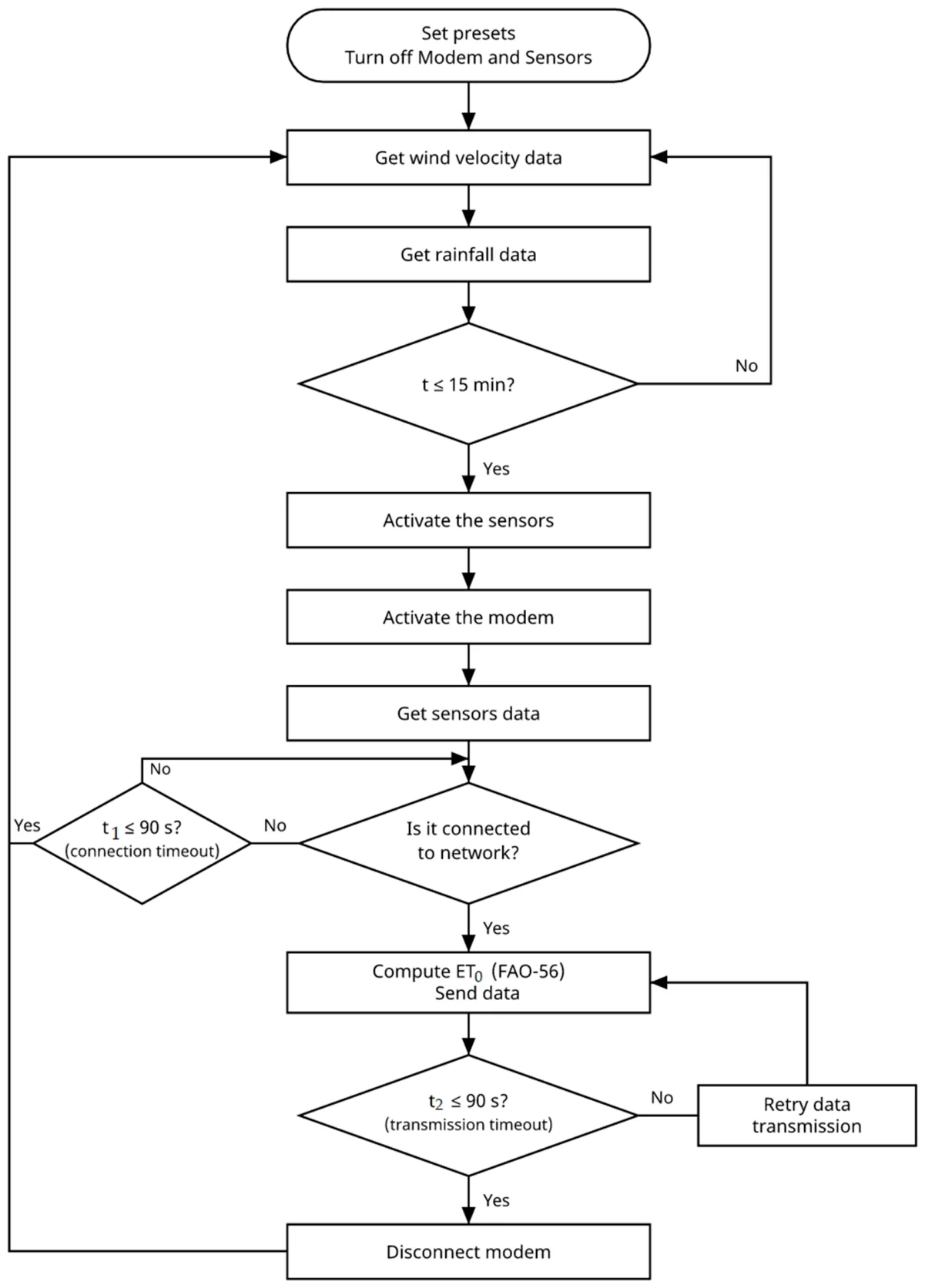
Operational workflow of the proposed IoF monitoring system. Environmental and soil measurements acquired by the sensing layer are digitized by the DAQ subsystem, processed by the ESP32 controller, encapsulated into JSON-formatted data packets, and transmitted to the application server via HTTP POST requests over the GSM/GPRS cellular network. The server validates incoming data, stores the measurements in a MySQL relational database, performs data processing and ET_0_ calculations, and delivers real-time information and decision-support services to web and mobile users.

After each acquisition cycle, the synchronized sensor signals are encapsulated into a standardized, low-overhead JSON-formatted payload. This dynamic data structure includes the station identifier, UTC timestamp, individual sensor metrics, power management telemetry (battery state-of-charge), and network link diagnostics. Utilizing cellular connectivity (GSM/GPRS) over standard HTTP POST requests, the IoF gateway transmits these JSON payloads to the central application server hosted at Akdeniz University, where incoming data streams are validated, parsed, and logged into a relational MySQL database for real-time visualization and irrigation decision support. The hardware interconnectivity and the underlying operational sequence of this acquisition pipeline are comprehensively illustrated in Figure 3 and Figure 4, respectively.

Furthermore, to balance high-frequency data capturing with power constraints, a strategic duty-cycle management protocol is employed: high-power peripherals (such as the cellular modem, camera module, and specialized sensors) are energized at discrete 15 min sampling intervals, whereas uninterrupted, event-driven sensors—namely wind speed and rainfall pulse counters—remain continuously active to ensure real-time event detection without data loss.

#### 2.2.3. Application Layer

**Figure 5:**
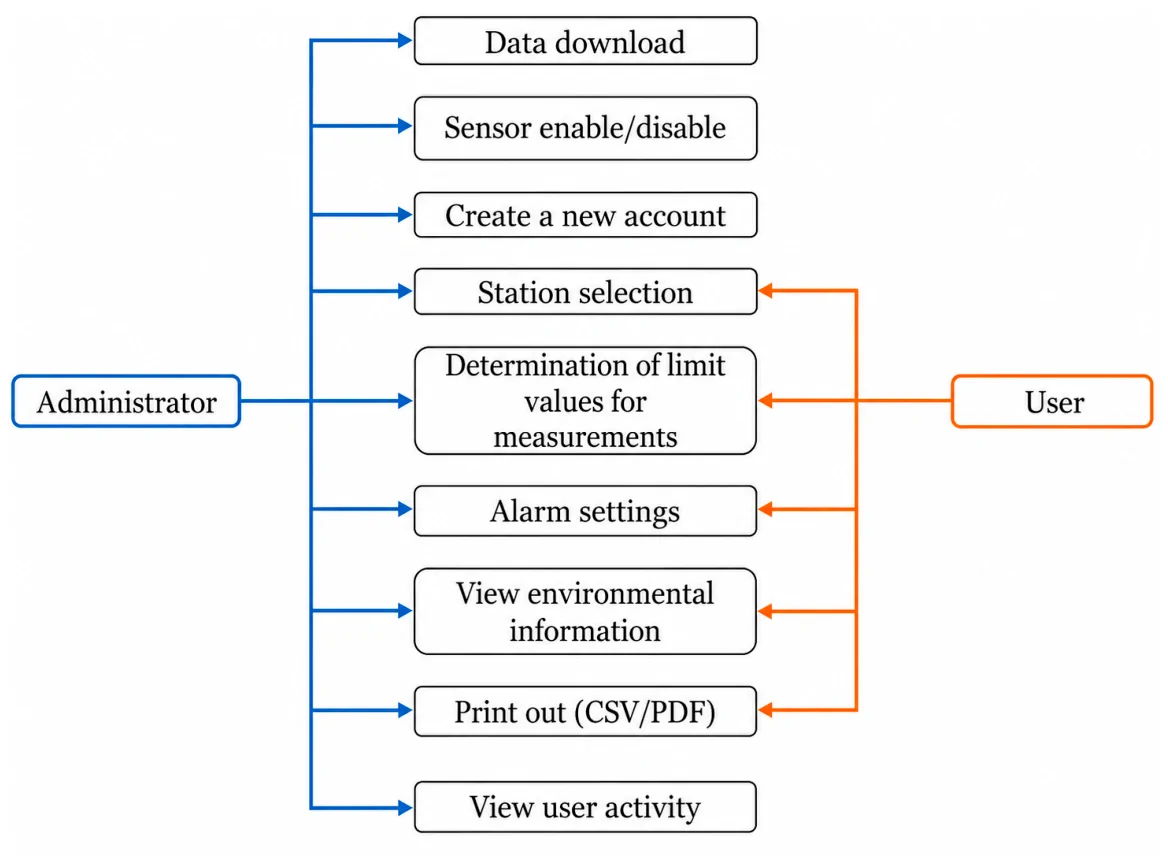
Software architecture of the proposed IoF platform illustrating the interaction between the embedded firmware, cloud-based data management system, database services, analytical modules, and graphical user interface. The software framework supports automated data acquisition, secure storage, historical data retrieval, environmental monitoring, reference evapotranspiration (ET_0_) estimation, and real-time irrigation management through web- and mobile-based applications.

The final layer involves a cloud-based infrastructure where the raw data, transmitted via the cellular modem, is processed and stored in structured electronic formats. This layer serves as the central hub for data management, supporting multi-platform accessibility through web and mobile interfaces. Within this framework, acquired parameters are visualized through interactive dashboards for real-time monitoring and subsequent statistical analysis of ET_0_ and ET_C_ values. To ensure system security and data integrity, the application layer incorporates robust user authentication with multiple permission levels, while the software backend employs a connection pooling strategy to minimize latency and ensure stable performance during high-volume data requests. The role-based access control logic of the cloud platform is illustrated in Figure 5. As depicted in the functional hierarchy, while the administrator role possesses full authority over system configurations, including sensor management, account creation, and threshold determination, the user role is restricted to operational functions such as data visualization and reporting.

Taken together, the perception, network, and application layers described above reflect a design rationale oriented not toward advancing sensor or communication engineering in isolation, but toward integrating these established technologies into a single platform expressly capable of contrasting localized and regional meteorological data. It is this integration, rather than any individual hardware or software component, that constitutes the specific contribution of the proposed architecture relative to prior IoF/IoT deployments in agriculture, which have not been designed to resolve spatial ET_0_ discrepancies of this kind or to translate them into agronomically actionable irrigation guidance for grapevine cultivation.

### 2.3. Data Integration and Irrigation Management Framework

Within the perception layer, climatic variables, soil parameters, and irrigation water quality are monitored in real-time under actual field conditions. These localized measurements are transmitted via the network layer to the application layer, where they are processed using the FAO Penman–Monteith method to derive precise daily ET_0_ values. Finally, this processed data is translated into actionable irrigation intelligence, enabling producers to perform site-specific evaluations of crop water requirements. This integrated approach not only supports more informed irrigation planning but also mitigates the operational risks associated with reliance on spatially averaged regional weather data.

### 2.4. Reference Evapotranspiration Estimation

Reference evapotranspiration (ET_0_, mm/day) was calculated using the FAO-56 Penman–Monteith equation [4,43], as expressed in Equation (1):

(1) $${\mathrm{ET}}_{0}\;=\frac{0.408\Delta \left(R_{n}\;-\;G\right)\;+\;\gamma \;\frac{900}{T\;+\;273}\;u_{2}\left(e_{s}\;-\;e_{a}\right)}{\Delta \;+\;\gamma \left(1\;+\;0.34u_{2}\right)}$$

where R_n_ is the net radiation at the crop surface (MJ/m^2^·day), G is the soil heat flux density (MJ/m^2^·day), which is typically neglected (G ≈ 0) for daily time steps as suggested by the FAO-56 protocol [4], T is the mean daily air temperature at 2 m height (°C), u_2_ is the wind speed at 2 m height (m/s), e_s_ is the saturation vapor pressure (kPa), e_a_ is the actual vapor pressure (kPa), e_s_ − e_a_ is the vapor pressure deficit (kPa), Δ is the slope of the saturation vapor pressure curve (kPa/°C), and γ is the psychrometric constant (kPa/°C).

The slope of the saturation vapor pressure curve (Δ) was determined as follows:

(2) $$\Delta \;=\;\frac{4098\;\left[0.6108\;\exp\;\left(\frac{17.27T}{T\;+\;237.3}\right)\right]}{{\left(T\;+\;237.3\right)}^{2}}$$

where T is the mean daily air temperature (°C), and Δ is the slope of the saturation vapor pressure curve (kPa/°C).

The psychrometric constant (γ) was calculated based on the atmospheric pressure (P, kPa):

(3) γ = 0.000665 · P

where P is the atmospheric pressure (kPa) and γ is the psychrometric constant (kPa/°C).

Given the field station’s elevation of 39 m, the atmospheric pressure (P) was assigned as 100.8 kPa [44]. To verify this simplification quantitatively, the psychrometric constant was computed for both stations using the FAO-56 formula γ = 0.000665 × P, with P = 100.8 kPa. Both sites yield γ = 0.0670 kPa/°C, an identity confirmed by the uniform values reported in Table 3. Even under conservative assumptions allowing for a modest inter-site elevation difference, the resulting change in γ would remain below 0.00010 kPa/°C (<0.1%), propagating to a change in ET_0_ of less than 0.002 mm/day—more than two orders of magnitude below the observed mean bias (MBE = −0.695 mm/day). The uniform γ assumption is therefore physically justified and exerts no material influence on any reported result.

Wind speed measurements obtained at a height of 3.5 m were adjusted to the standard 2 m height (u_2_) to ensure compatibility with the FAO-56 Penman-Monteith equation. The following logarithmic wind profile relationship was employed for this adjustment:

(4) $$u_{2\;}=\;u_{z}\frac{4.87}{\ln(67.8z\;-\;5.42)}$$

where u_z_ is the measured wind speed (m/s) at height (3.5 m), *z* is the measurement height above ground (m), and *u_2_* is the adjusted wind speed at the standard 2 m reference height (m/s). Given the station’s anemometer height, a conversion factor of 0.894 [4] was applied to the raw data to derive the reference wind speed values used in ET_0_ calculations.

The saturation vapor pressure (e_s_) was computed from the mean daily air temperature (T), and the actual vapor pressure (e_a_) was derived from e_s_ and the mean daily relative humidity (RH, %) as follows:

(5) $$e_{s}=0.6108exp\left(\frac{17.27T}{T+237.3}\right)$$

(6) $$e_{a\;}=e_{s}\times \frac{\mathrm{RH}}{100}$$

where e_s_ is the saturation vapor pressure (kPa), *T* is the mean daily air temperature (°C), e_a_ is the actual vapor pressure (kPa) and RH is the mean daily relative humidity (%).

Crop water consumption (ET_C_) was determined using the crop coefficient approach [4,45]:

(7) $${ET}_{c}=K_{c}\;\cdot \;{ET}_{o}$$

where K_C_ is the crop coefficient. For grapevine, K_C_ values were adopted from FAO-56 guidelines [4], assigned to the 2021 growing season months based on the phenological calendar applicable to the Mediterranean climate of Antalya. Specifically, April was assigned to the initial stage (K_C_ = 0.30), May to the crop development stage (K_C_ = 0.50), June and July to the mid-season stage (K_C_ = 0.70), August to the late-season stage (K_C_ = 0.55), and September to the late-season stage (K_C_ = 0.45). It is acknowledged that these tabulated values represent generalized estimates for surface irrigation conditions and may not fully reflect the specific cultivar characteristics, canopy architecture, or irrigation method employed at the study site; this is addressed as a limitation in Section 4.5.

### 2.5. Statistical Analysis

To quantify the discrepancy between field station-based and TSMS-based ET_0_ estimates, a robust statistical framework was implemented. In this framework, the ET_0_ values derived from the on-site field station were treated as the observed (reference) values (O_i_), whereas the TSMS-based ET_0_ data were evaluated as the predicted variables (P_i_). The analysis proceeded as follows:

- Comparative significance testing: A paired-sample *t*-test was employed to assess the statistical significance of the mean differences between the two ET_0_ time series at a significance level of α = 0.05. Given the relatively small sample size (n = 12 monthly means), however, the normality of paired differences cannot be assumed with confidence, and the central limit theorem provides insufficient justification at this scale. Accordingly, the Wilcoxon signed-rank test is treated as the primary inferential basis in this study, as it requires no distributional assumption and is robust to departures from normality. The paired *t*-test is retained as a complementary parametric reference consistent with precedent in the agrometeorological literature, but inferential conclusions are drawn principally from the nonparametric result.
- Error metrics: The absolute and relative discrepancies between the datasets were quantified using a suite of error metrics. Root Mean Square Error (RMSE), Mean Absolute Error (MAE), Mean Bias Error (MBE), and Mean Absolute Percentage Error (MAPE) were calculated using the following equations [46,47,48,49]:
  (8) $$RMSE=\sqrt{\frac{1}{n}\sum_{i=1}^{n}{\left(P_{i}-O_{i}\right)}^{2}}$$
  where Pi is the TSMS-based ET_0_ estimate for observation i (mm/day), Oi is the field station-based ET_0_ estimate for observation i (mm/day), and n is the number of observations.
  (9) $$\mathrm{MAE}=\frac{1}{n}\sum_{i=1}^{n}\left|P_{i}-O_{i}\right|$$
  (10) $$\mathrm{MBE}=\frac{1}{n}\sum_{i=1}^{n}\left(O_{i}-P_{i}\right)$$
  where Oi is the field station-based ET_0_ (observed), Pi is the TSMS-based ET_0_ (predicted), and *n* is the number of observations. A positive MBE indicates that field station ET_0_ systematically exceeds TSMS-based estimates on an annual mean basis.
  (11) $$MAPE=\frac{100\%}{n}\sum_{i=1}^{n}\left|\frac{O_{i}-P_{i}}{P_{i}}\right|$$
  where Oi is the field station-based ET_0_, Pi is the TSMS-based ET_0_ (reference denominator), and n is the number of observations.
- Correlation and agreement indices: The linear association between the data sources was evaluated using the Pearson correlation coefficient (r) and the coefficient of determination (R^2^). To further verify the predictive performance and convergence, the Nash–Sutcliffe Efficiency (NSE) and Willmott’s Index of Agreement (d) were computed [50,51,52,53]:
  (12) $$\mathrm{NSE}=1-\left[\frac{\sum_{i=1}^{n}{\left(O_{i}-P_{i}\right)}^{2}}{\sum_{i=1}^{n}{(O_{i}-\bar{O})}^{2}}\right]$$
  where Oi is the field station-based ET_0_ for observation i (mm/day), Pi is the TSMS-based ET_0_ for observation i (mm/day), Ō is the mean of all field station ET_0_ observations (mm/day), and n is the number of observations. NSE ranges from −∞ to 1; a value of 1 indicates perfect agreement, and a value below 0 indicates that the mean of observed values is a better predictor than the model.
  (13) $$d=1-\left[\frac{\sum_{i=1}^{n}{\left(P_{i}-O_{i}\right)}^{2}}{\sum_{i=1}^{n}{\left(\left|P_{i}-\bar{O}\right|+\left|O_{i}-\bar{O}\right|\right)}^{2}}\right]$$
  where Pi is the TSMS-based ET_0_, Oi is the field station-based ET_0_, Ō is the mean of observed values, and n is the number of observations. Values of d range from 0 to 1, with 1 indicating perfect agreement between observed and predicted values.

The Coefficient of Variation (CV) for both Field (CV_Field_) and TSMS (CV_TSMS_) data was calculated to assess the relative variability [54,55]:

(14) $$\mathrm{CV}\left(\%\right)=\left(\frac{\sigma }{\mu }\right)\times 100$$

where *σ* is the standard deviation and *μ* is the mean of the respective dataset (field station or TSMS). CV is expressed as a percentage and quantifies the relative dispersion of each data source:

- Deviation modeling: Linear regression analysis was performed to characterize the relationship between ET_0_ deviation (ΔET_0_ = ET_0,Field_ − ET_0,TSMS_) and key meteorological drivers, with a specific focus on air temperature and solar radiation.
- Irrigation scenario analysis: To evaluate the practical implications for precision viticulture, seasonal crop evapotranspiration (ET_C_) was estimated by applying both data sources. By integrating stage-specific crop coefficients (K_C_) for grapevine—incorporating initial (0.30), development (0.50), mid-season (0.70), and late-season (0.45–0.55) stages—the volumetric water differences were quantified.

All statistical metrics—including Pearson r, R^2^, the paired *t*-test, RMSE, MAE, MBE, MAPE, NSE, Willmott’s d, and CV—were derived from monthly means (n = 12), consistent with the FAO-56 crop coefficient-based irrigation scheduling framework that operates at monthly timescales. The linear regression characterizing the relationship between ET_0_ deviation (ΔET_0_) and mean air temperature was likewise computed from monthly means (n = 12), ensuring methodological consistency across all analyses. While monthly aggregation aligns the statistical framework with the operational resolution of FAO-56 irrigation scheduling, results should be interpreted in the context of this temporal scale, and daily resolution analyses in future studies would provide a complementary perspective on the short-term dynamics of ET_0_ discrepancies.

The percentage differences (Δ%) presented in Table 4 were calculated relative to the TSMS-based ET_0_ estimate, as follows:

(15) $$\Delta \left(\%\right)\;=\;\frac{\left|O_{i}\;-\;P_{i}\right|}{P_{i}}\times 100$$

where O_i_ is the field station-based ET_0_ for observation i (mm/day) and Pᵢ is the TSMS-based ET_0_ for observation i (mm/day). The absolute value ensures that Δ% represents the magnitude of deviation regardless of direction. Monthly values reported in Table 4 correspond to Δ% calculated on a monthly basis (n = 12), without averaging daily values [56].

## 3. Results

### 3.1. System Performance and Data Acquisition

The monitoring system demonstrated robust operational stability throughout the observation period, ensuring the continuous real-time acquisition of critical agro-meteorological and edaphic parameters—including air temperature, relative humidity, solar radiation, wind dynamics, atmospheric pressure, and precipitation, alongside sub-surface soil conditions and irrigation water quality metrics such as pH and electrical conductivity (EC). The monitoring campaign spanned a full calendar year to capture complete seasonal variability. Data acquisition reached a completeness rate of 98.6%, ensuring that the derived monthly representative values (n = 12) for (ET_0_ and other parameters remained highly accurate despite 5 days of minor connectivity-related gaps. The five days of missing data, attributable solely to intermittent GSM/GPRS connectivity failures, were non-consecutive and distributed across the monitoring period. Missing daily records were excluded from monthly mean calculations rather than interpolated, in accordance with standard agrometeorological data management practice. Given that all ET_0_ and ETc analyses were conducted on monthly means (n = 12), the exclusion of fewer than two days per month has a negligible effect on the reported aggregates and does not materially influence the comparative conclusions of this study. Sensor readings were compared against TSMS records as a standardised regional benchmark for temperature and humidity during the overlapping measurement period; mean absolute deviations remained within the manufacturer-specified accuracy thresholds (±0.5 °C for temperature, ±2% for relative humidity), confirming the internal consistency of the field station data used in subsequent ET_0_ calculations. The cloud-integrated user interface facilitated advanced data management through a web-based platform where users could access these recorded parameters. Upon selecting the desired variables, the interface provided comprehensive access to real-time, average, and historical measurement results.

**Figure 6:**
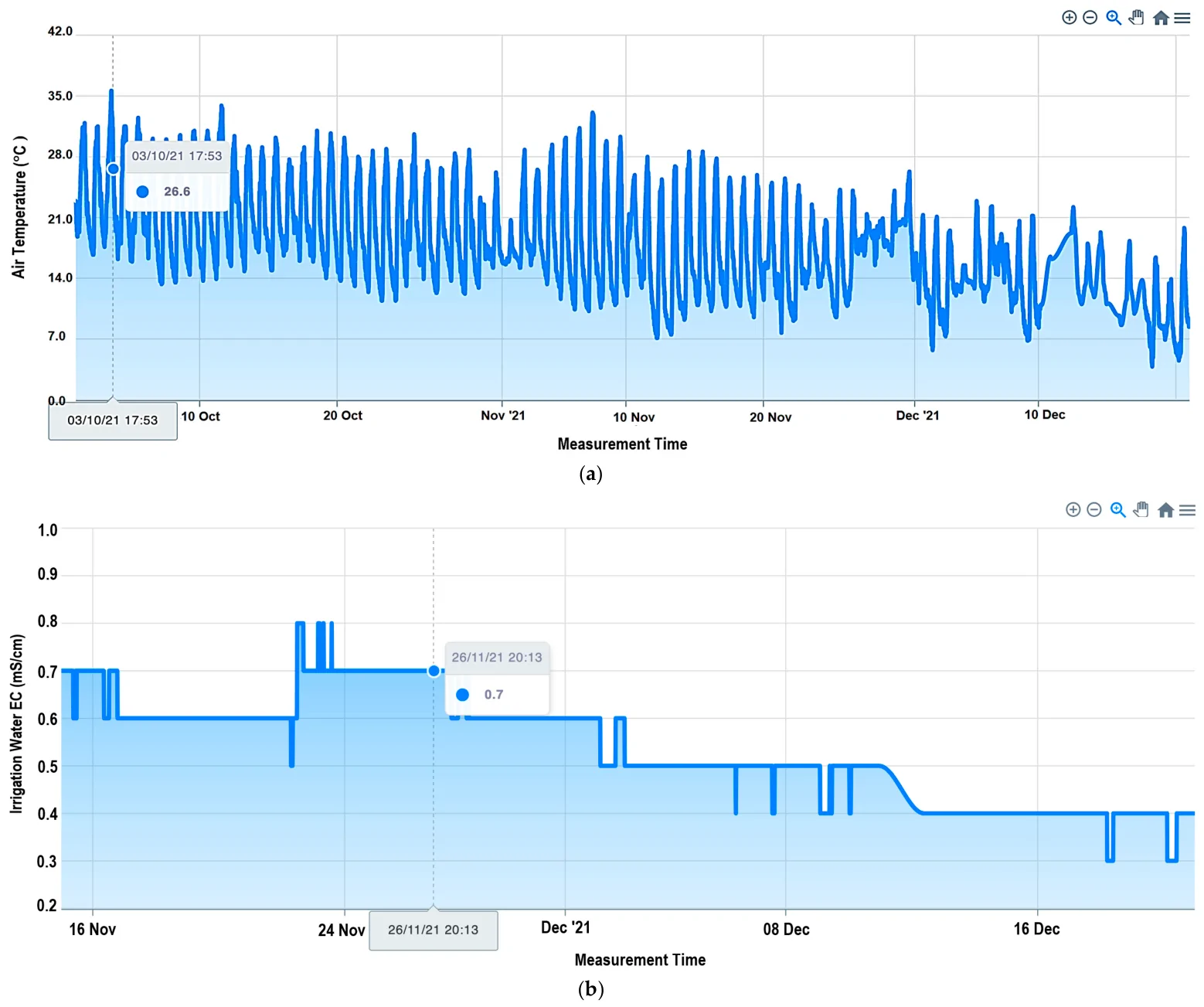
Representative panels of the cloud-based IoF monitoring dashboard: (**a**) air temperature (°C) and (**b**) irrigation water electrical conductivity (mS/cm) time-series records acquired during the 2021 monitoring period. Additional dashboard panels displaying soil temperature and global solar radiation records are provided in Figure S1 of the Supplementary Materials.

The analytical strength of the platform was further highlighted by its ability to generate high-resolution graphical visualizations for longitudinal performance analysis. As illustrated in Figure 6a,b, presenting a representative high-resolution data segment from the 2021 monitoring period, the interface enables direct monitoring of the vineyard’s thermal regime and irrigation water quality. Additional dashboard panels displaying soil temperature dynamics and global solar radiation records are provided in Figure S1 of the Supplementary Materials. Beyond these graphical representations, the system allowed users to view phenological images of the plants captured at recorded intervals, facilitating remote observation and documentation of crop development across distinct phenological transitions.

To support timely and effective interventions, the platform incorporated an automated early warning system. Producers could define specific threshold values to trigger alarm states for adverse conditions such as frost or thermal stress. In the event of an alarm, instant notifications were sent to designated mobile phones and email addresses, enabling timely and responsive field intervention.

### 3.2. Comparative Analysis and Benchmark Comparison with Official Meteorological Data (TSMS)

**Figure 7:**
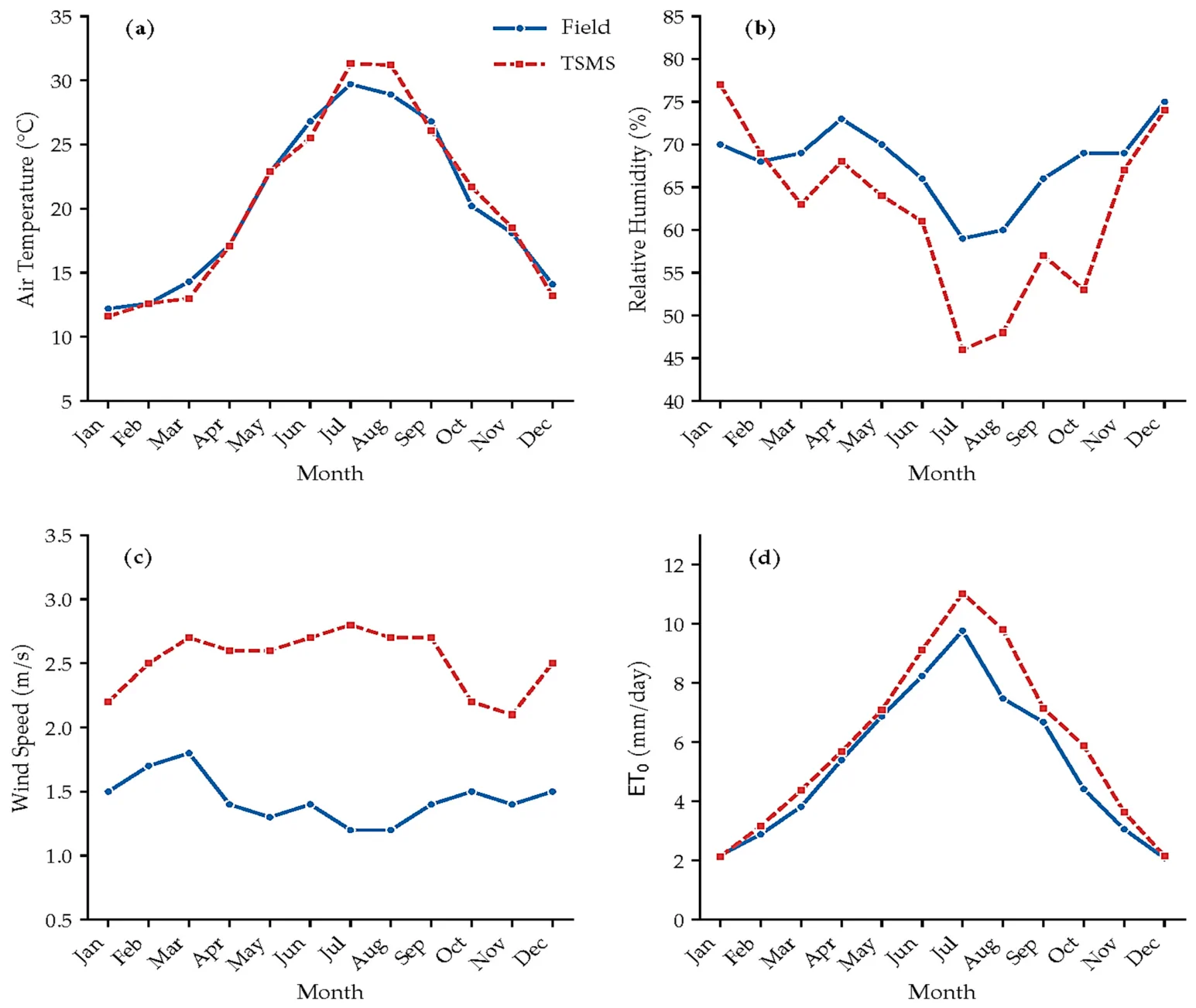
Monthly comparison of key meteorological variables between the on-site field station and TSMS records for the 2021 calendar year: (**a**) mean air temperature (°C), (**b**) relative humidity (%), (**c**) wind speed at 2 m height (m/s), and (**d**) calculated reference evapotranspiration (ET_0_, mm/day).

Prior to evaluating discrepancies in ET_0_ estimations, the underlying meteorological inputs from the on-site field station (Table 3) were compared against TSMS datasets to verify sensor reliability. Figure 7 presents a multi-panel comparative analysis of air temperature, relative humidity, wind speed, and the subsequent monthly ET_0_ estimates. By utilizing synchronized daily observation averages from both the localized IoF field station and the TSMS records, this comparative framework serves to benchmark in situ IoF sensing capabilities against a quality-controlled regional meteorological reference and highlights the profound impact of microclimatic variations on irrigation requirement modeling.

The analysis revealed that while air temperature showed close agreement during the transitional months, significant divergence emerged during the summer season. Notably, the field station recorded a monthly mean of 28.9 °C in August, whereas the TSMS reported a markedly higher value of 31.2 °C for the same period—a difference of 2.3 °C attributable to the urban heat island effect at the regional station. Furthermore, relative humidity was consistently higher at the experimental site throughout most of the year. This phenomenon is likely intensified by the vineyard canopy, which facilitates evaporative cooling; for instance, the TSMS recorded substantially lower humidity in July compared to the field station (46% vs. 59%). Conversely, wind speed was systematically lower at the field station (range: 1.2–1.8 m/s) relative to TSMS records (range: 2.1–2.8 m/s), a disparity attributable to the sheltering effect of the vineyard vegetation and local topographical shielding.

These variations are primarily driven by microclimatic heterogeneity stemming from the geographical distance between the experimental site and the official meteorological station. Additionally, topographical differences and the urban heat island (UHI) effect at the TSMS site [57,58]—exacerbated by increasing urbanization surrounding the station—were identified as critical drivers of these discrepancies. At the TSMS location, the absence of vegetative cover suppresses nocturnal radiative cooling and maintains artificially elevated temperatures, particularly during summer months.

It is important to distinguish between two conceptually distinct sources of inter-station divergence observed in this study. The first is instrument difference, which refers to measurement uncertainty attributable to sensor type, calibration status, and manufacturer-specified accuracy thresholds. The second, and substantially more consequential, is site-exposure difference, which reflects the contrasting surface boundary conditions governing the microclimate at each location. The field station is embedded within an actively transpiring vineyard canopy, where vegetative shading, evaporative cooling, and aerodynamic sheltering systematically reduce air temperature, increase relative humidity, and attenuate wind speed relative to open-exposure conditions. The TSMS station, by contrast, is situated in an urban environment approximately 15 km distant, where impervious surfaces, the absence of vegetative cover, and anthropogenic heat fluxes sustain the urban heat island effect, suppress nocturnal radiative cooling, and maintain artificially elevated temperatures and wind exposure. The meteorological divergences documented in Table 3, particularly the systematic differences in wind speed (Δu_2_ = −1.08 m/s annual mean), relative humidity (ΔRH = +5.6% annual mean), and summer air temperature (e.g., August: ΔT = −2.3 °C), are therefore attributable primarily to site-exposure differences rather than instrument uncertainty, as confirmed by the cross-validation of sensor readings against TSMS records, which demonstrated mean absolute deviations within manufacturer-specified accuracy thresholds (±0.5 °C for temperature, ±2% for relative humidity). This distinction is agronomically significant: it implies that even a perfectly calibrated instrument deployed at the TSMS location would yield ET_0_ estimates that diverge systematically from field-level values, because the surface energy and mass balance conditions governing evapotranspiration at the two sites are structurally different.

**Figure 8:**
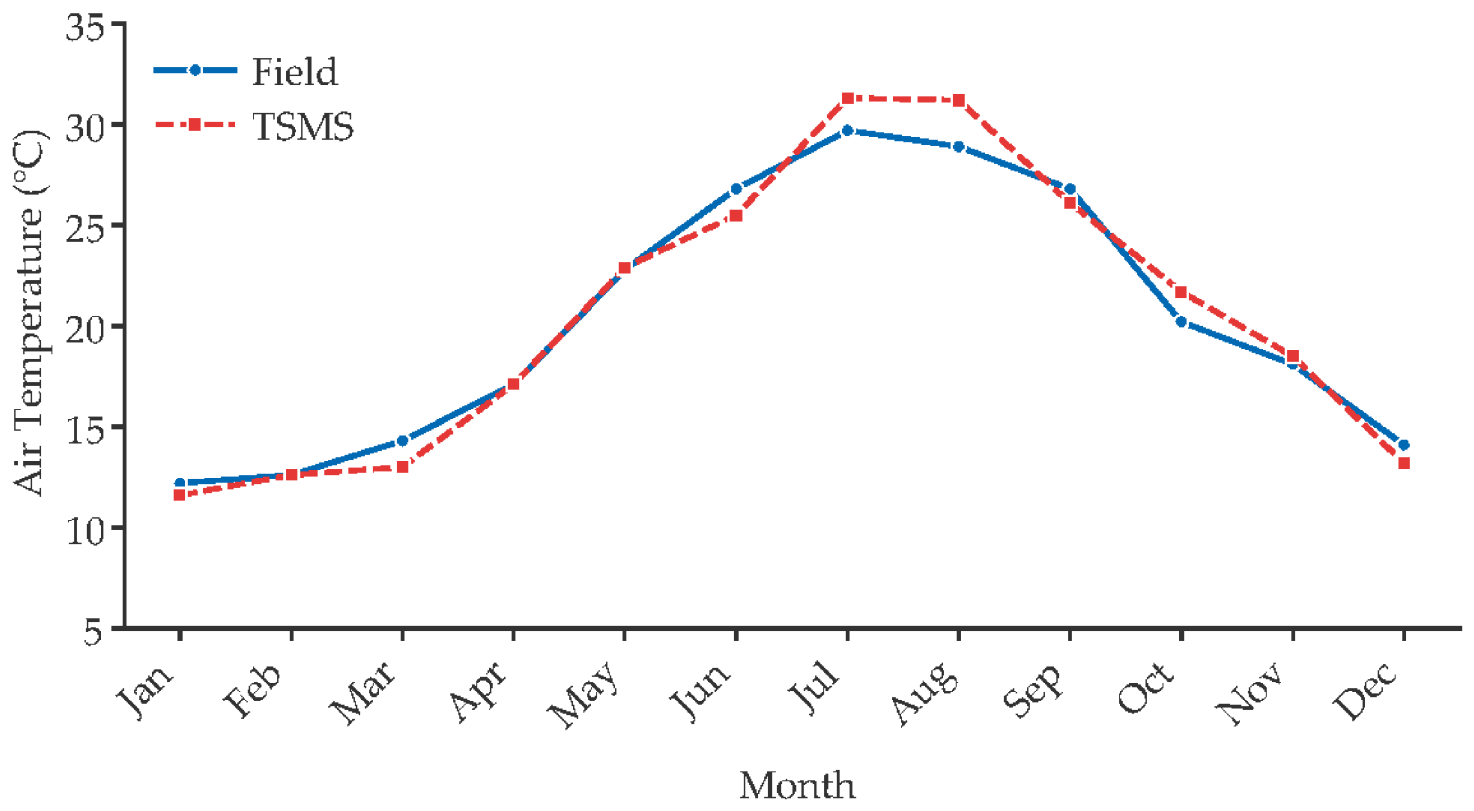
Monthly mean air temperature records from the TSMS regional station and the on-site field station throughout the 2021 monitoring period. Deviations between the two sources are most pronounced in August and October, reflecting the combined influence of the urban heat island effect at the TSMS site and site-specific microclimatic conditions—including vegetative shading and topographical positioning—at the vineyard.

To evaluate the field station data within the context of regional meteorological conditions, Figure 8 presents the TSMS temperature records alongside the corresponding field station measurements for the 2021 monitoring period.

While the field station and TSMS temperature records followed broadly consistent seasonal trends throughout the 2021 monitoring period, site-specific divergences were most apparent during the summer months. In August, the field station recorded a monthly mean of 28.9 °C compared to 31.2 °C at the TSMS station, a difference of 2.3 °C attributable to the cooling influence of active crop evapotranspiration within the vineyard canopy (the oasis effect) [59]. In October, both stations recorded similar values, with the TSMS slightly elevated (21.7 °C) relative to the field station (20.2 °C), consistent with reduced vegetative cover and greater exposure to sensible heat fluxes at the regional station. Together, these divergences illustrate the spatial heterogeneity of the temperature field and its sensitivity to prevailing surface energy balance conditions.

### 3.3. Reference Evapotranspiration: Descriptive Comparison

Monthly ET_0_ values calculated from both data sources are summarized in Table 4, with detailed distribution characteristics provided in Table 5. Field station-based ET_0_ estimates ranged from 2.09 mm/day (December) to 9.77 mm/day (July), with an annual mean of 5.24 mm/day. TSMS-based estimates exhibited a broader range, from 2.14 mm/day (January) to 11.03 mm/day (July), with an annual mean of 5.94 mm/day. TSMS-based estimates consistently exceeded field station values across nearly all months, with the sole exception of January, where the two sources were essentially equivalent (Δ = +0.04 mm/day). The most pronounced absolute deviations occurred in August (Δ = −2.32 mm/day; 23.7%), October (Δ = −1.47 mm/day; 25.0%), and July (Δ = −1.26 mm/day; 11.4%).

### 3.4. Statistical Analysis of ET_0_ Discrepancies

A comprehensive suite of statistical tests was applied to quantify the agreement between field station-based and TSMS-based ET_0_ estimates. The comparative results, summarized in Table 5, reveal systematic discrepancies that have significant implications for precision water management.

The analysis demonstrates that both datasets exhibit broadly comparable seasonal variability, with a CV of 50.26% for TSMS and 48.66% for the field station (Table 5). This similarity in relative dispersion indicates that the systematic discrepancy between the two sources manifests primarily as a consistent additive bias rather than a difference in seasonal amplitude.

The Wilcoxon signed-rank test (W = 1, *p* < 0.001) provides the more robust inferential confirmation of systematic ET_0_ overestimation by TSMS, given the sample size constraints associated with monthly aggregation (n = 12). The paired *t*-test result (t = −3.51, *p* = 0.005) is consistent with this finding and is reported for comparability with the broader agrometeorological literature, where parametric tests remain the prevailing convention. Consequently, these sources cannot be considered interchangeable for precision irrigation applications. This statistical divergence is rooted in opposing seasonal biases: while the field station yields higher ET_0_ values during cool months, it records lower values during warm months. The magnitude of these fluctuations is sufficiently asymmetric to produce a statistically detectable annual mean difference.

**Figure 9:**
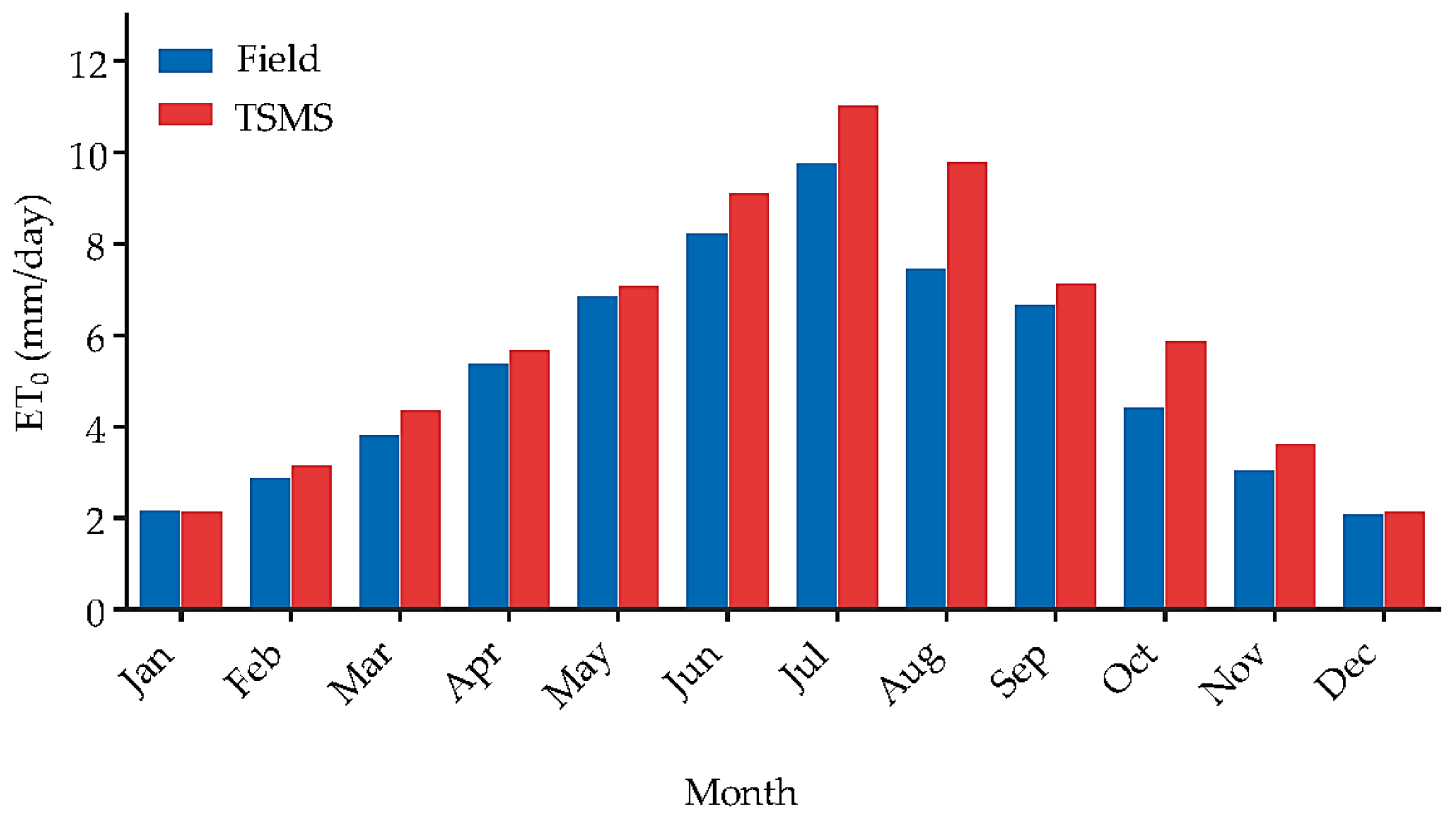
Monthly ET_0_ estimates (mm/day) derived from the on-site field station and TSMS data for the 2021 annual monitoring period. Both datasets exhibit comparable seasonal variability (CV_Field_ = 48.66%; CV_TSMS_ = 50.26%); however, TSMS-based estimates consistently exceed field station values, with the overestimation intensifying during the peak summer months.

The high Pearson correlation (r = 0.9814) and R^2^ = 0.9631 confirm that both datasets share strongly coherent seasonal dynamics. The Nash–Sutcliffe Efficiency (NSE = 0.8466) indicates very good predictive correspondence, and Willmott’s index of agreement (d = 0.9679) reflects near-perfect seasonal synchronization between the two sources. Together, these indices reveal that the discrepancy between the datasets manifests as a systematic, directionally consistent overestimation by TSMS rather than random disagreement. Despite this high correlation, the statistically significant mean bias (MBE = −0.695 mm/day) and the systematic departure from the 1:1 correspondence line confirm that TSMS data cannot be used as an unmodified substitute for site-specific irrigation scheduling, as further illustrated in Figure 9.

### 3.5. Seasonal Pattern and Driving Variables

**Figure 10:**
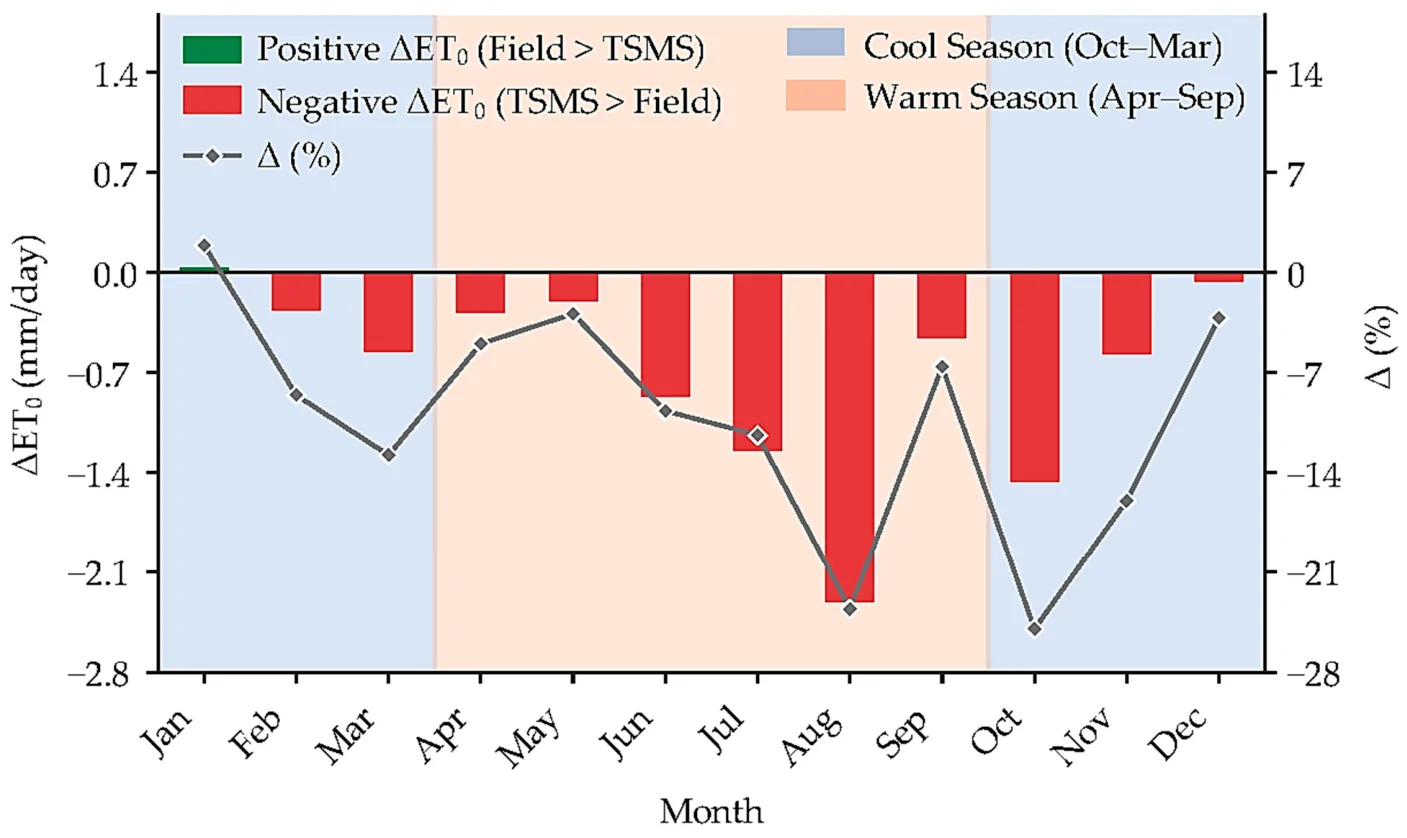
Monthly ET_0_ deviation (ΔET_0_ = ET_0_,Field − ET_0_,TSMS, mm/day) and corresponding relative percentage differences. Positive values (green bars) indicate months in which field station ET_0_ exceeds TSMS estimates; negative values (red bars) indicate the reverse. Blue and pink shaded regions denote the cool season (October–March) and warm season (April–September), respectively.

A pronounced temperature-dependent pattern was observed in the magnitude of ET_0_ deviation. Figure 10 illustrates the monthly ΔET_0_ (Field station − TSMS) values, with positive deviations (green) indicating months where the field station recorded higher ET_0_ than TSMS and negative deviations (red) indicating TSMS overestimation. TSMS overestimated field-level ET_0_ in 11 of 12 months, with the single near-zero exception occurring in January (ΔET_0_ = +0.04 mm/day), demonstrating a consistent directional bias rather than a seasonal reversal.

**Figure 11:**
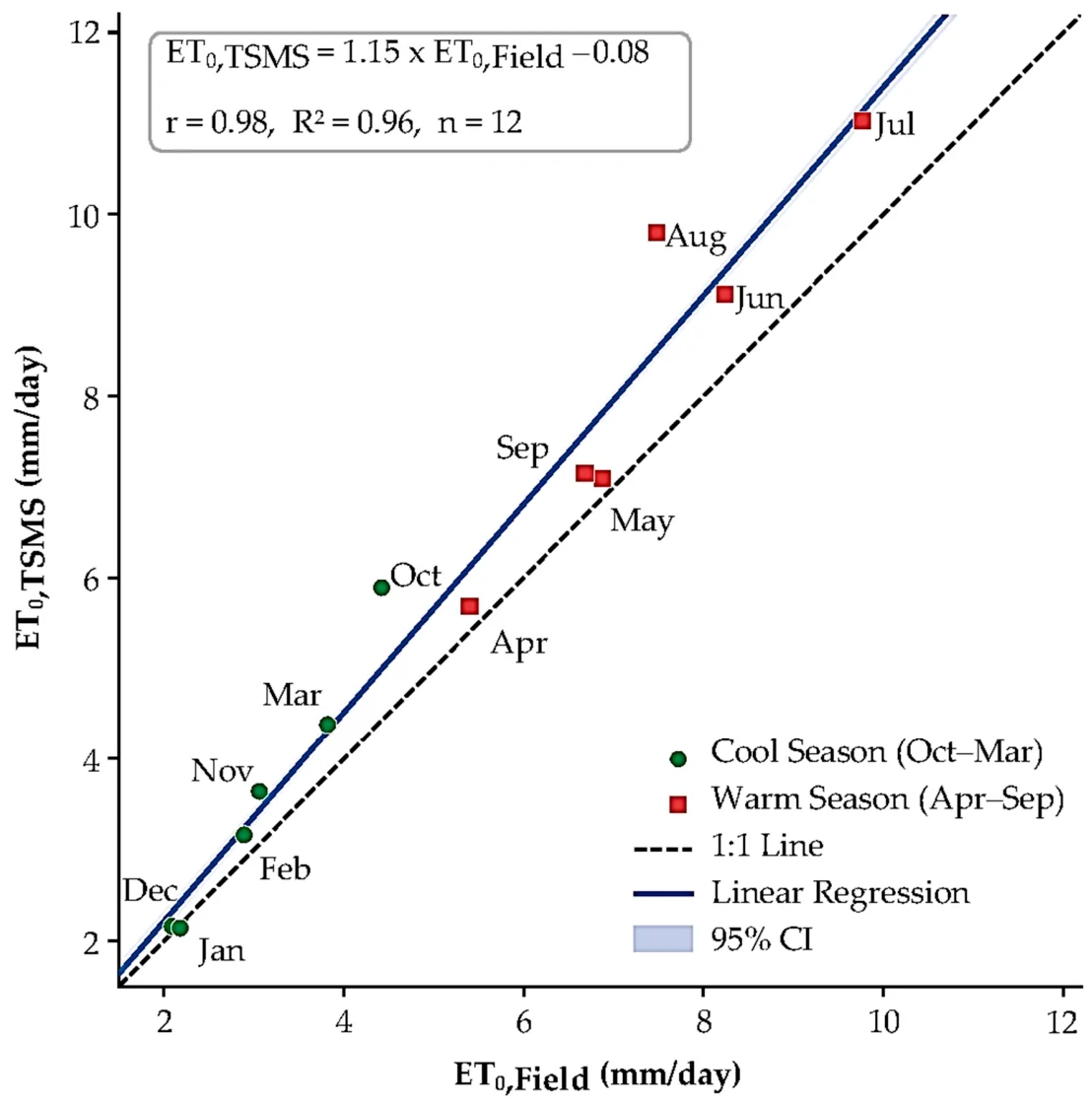
Comparison of monthly TSMS-based versus field station-based ET_0_ estimates for the 2021 monitoring period, including the 1:1 line of perfect agreement and the linear regression fit (ET_0_,TSMS = 1.15 × ET_0_,Field − 0.08; r = 0.98, n = 12). Green and red markers denote cool-season (October–March) and warm-season (April–September) months, respectively.

The relationship between TSMS-based and field station-based ET_0_ is illustrated in Figure 11, where monthly means are compared against a 1:1 line of perfect agreement and a linear regression fit (ET_0_,TSMS = 1.15 × ET_0_,Field − 0.08; r = 0.98, n = 12); the systematic departure from the 1:1 line further confirms that TSMS data cannot be used as a direct substitute for localized field measurements. Consistent with this finding, TSMS-based estimates exceeded field station values in 11 of 12 months throughout the monitoring period. The TSMS overestimation was smallest during the winter months (January: Δ = +0.04 mm/day, 1.9%; December: Δ = −0.07 mm/day, 3.2%) and intensified progressively through the growing season, reaching its maximum in August (Δ = −2.32 mm/day; 23.7%) and October (Δ = −1.47 mm/day; 25.0%). Statistical performance metrics for this comparison are further detailed in the upper-left inset of the figure.

**Figure 12:**
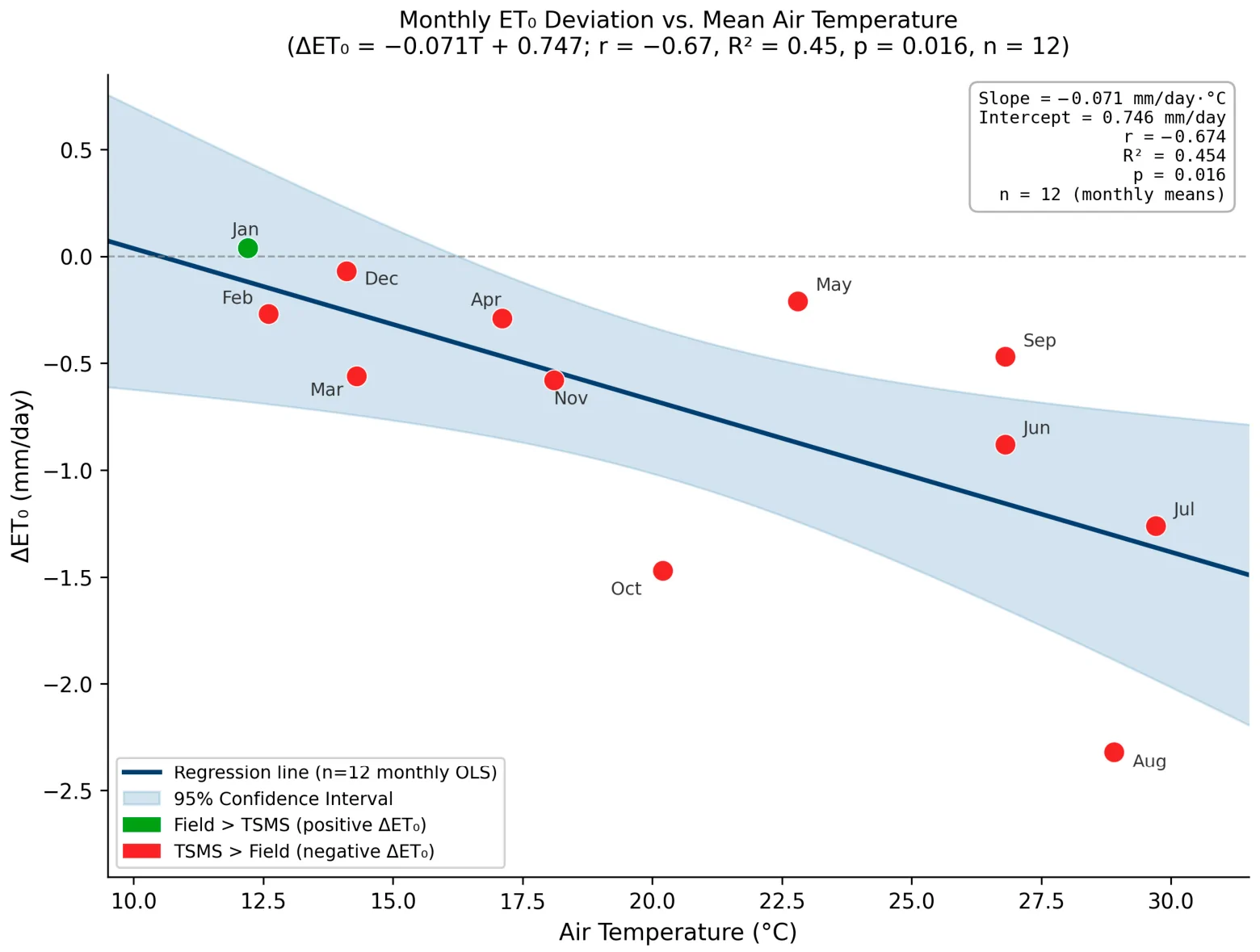
Monthly ET_0_ deviations (ΔET_0_) as a function of mean air temperature (T). The regression line includes a 95% confidence interval (shaded area); marker colors indicate the direction of deviation (green: field > TSMS; red: TSMS > field). The dashed horizontal line denotes zero deviation (ΔET_0_ = 0), separating months in which TSMS overestimated field-level ET_0_ (below the line) from those in which it underestimated it (above the line).

Linear regression of monthly means (n = 12) revealed a significant inverse relationship between mean monthly air temperature and ET_0_ deviation (Figure 12): ΔET_0_ = −0.071T + 0.747 (r = −0.67, R^2^ = 0.45, *p* = 0.016). This confirms a statistically significant, temperature-indexed bias structure—whereby air temperature serves as a seasonal index for the integrated intensification of wind speed, relative humidity, and net radiation differences between the two sites, rather than as the dominant direct driver of ET_0_ discrepancy; the attribution analysis (Table 6) confirms that the direct contribution of air temperature differences remains modest (~2.4%), while wind speed (~36.6%), net radiation (~31.0%), and relative humidity (~30.0%) constitute the primary physical pathways. The physical drivers of this bias are further elucidated through the variable-by-variable attribution analysis presented in Table 6 and discussed in Section 4.2. The regression explains 45% of the variance in monthly ET_0_ deviation, confirming a statistically significant temperature-dependent bias structure. No crossover temperature is reported from this analysis, as the model intercept corresponds to a temperature of approximately 10.5 °C—below the minimum observed monthly mean of 12.2 °C—placing the zero-crossing outside the range of observed data and therefore beyond reliable interpretation. In practice, TSMS overestimated field-level ET_0_ in 11 of 12 months throughout the monitoring period, with only January approaching near-zero deviation (ΔET_0_ = +0.04 mm/day). In contrast, solar radiation did not show a significant relationship with ET_0_ deviation (r = −0.23, *p* = 0.472), consistent with the interpretation that both sites receive broadly comparable incoming radiation but differ substantially in surface energy partitioning owing to differences in land cover and vegetation.

To further elucidate the physical drivers of the observed ET_0_ discrepancy, a variable-by-variable attribution analysis was conducted by substituting each TSMS input individually into the FAO-56 Penman–Monteith equation while holding all other inputs at field station levels (Table 6). This analysis reveals that wind speed differences (Δu_2_ = −1.08 m/s annual mean) account for approximately 36.6% of the total explainable ET_0_ discrepancy, with relative humidity differences (ΔRH = +5.6% annual mean) and net radiation differences (ΔRn) contributing approximately 30.0% and 31.0%, respectively. The direct contribution of air temperature differences (ΔT = −0.09 °C annual mean) was modest at 2.4%, reflecting the fact that the annual mean temperature difference between the two stations is small. These proportions were consistent across seasons, with wind speed and relative humidity contributions intensifying during the warm season (April–September), when the vineyard canopy effect and the TSMS urban boundary layer diverge most strongly. The strong inverse relationship between T_Field_ and ΔET_0_ (r = −0.67) therefore reflects the role of field station air temperature as a *seasonal index* for the integrated microclimate state of the vineyard—capturing the combined seasonal amplification of humidity, wind, and radiation differences—rather than a direct causal pathway. This interpretation is consistent with the attribution results and provides the physical basis for the regression equation ΔET_0_ = −0.071T + 0.747 as a practical correction tool.

### 3.6. Irrigation Impact Assessment

**Figure 13:**
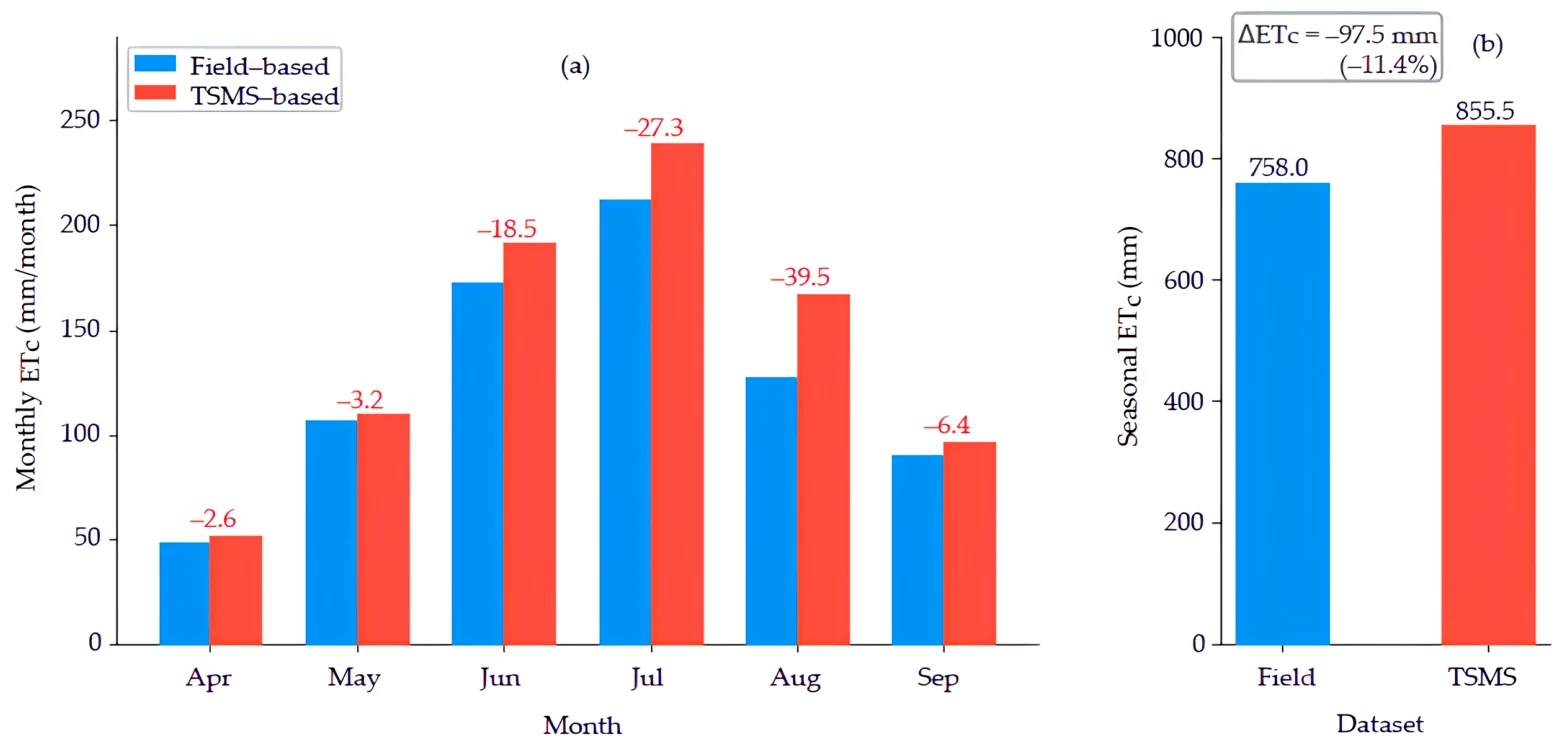
Comparison of grapevine crop evapotranspiration (ET_C_) estimates derived from field station and TSMS data for the growing season (April–September 2021): (**a**) monthly ET_C_ values (mm/month) from both data sources, with annotated absolute differences (mm/month) for each month; (**b**) cumulative seasonal ET_C_ totals, illustrating the overall water requirement difference between the two data sources.

To translate ET_0_ deviations into practical irrigation consequences, a scenario analysis was conducted for the grapevine growing season (April–September). Monthly crop evapotranspiration (ET_C_) was estimated by applying FAO-56 stage-specific crop coefficients (K_C_), aligned with the Mediterranean phenological calendar [4], to both field and TSMS-derived ET data. The results, summarized in Table 7 and illustrated in Figure 13, reveal a consistent overestimation of crop water requirements when using regional station data. Specifically, the seasonal cumulative discrepancy reached 97.5 mm, representing an 11.4% deviation relative to the TSMS-derived seasonal total (855.5 mm).

As evident from Table 7, the direction of the ET_C_ discrepancy follows a clear seasonal gradient aligned with the temperature-dependent bias structure identified in Section 3.5. TSMS-based ET_0_ exceeded field station estimates throughout the entire growing season, with the magnitude of overestimation increasing with temperature. During the early vegetative period (April–May), TSMS overestimated ET_C_ by 2.6 mm (5.0%) in April and 3.2 mm (2.9%) in May. During the peak summer months, the overestimation intensified substantially: TSMS-based ET_C_ exceeded field station estimates by 18.5 mm in June (9.7%), 27.3 mm in July (11.4%), and 39.5 mm in August (23.7%), the latter representing the largest single-month discrepancy of the growing season. A smaller but agronomically relevant overestimation of 6.4 mm (6.6%) was recorded in September. This progressively increasing divergence with rising seasonal temperatures statistically substantiates the agronomic case for IoF-based localized sensing over regional meteorological data as the primary input for irrigation decision-support systems.

Comparative analysis indicates that utilizing TSMS-derived data results in a cumulative seasonal ET_C_ estimate of 855.5 mm, whereas localized field station measurements yield a lower value of 758.0 mm. This represents a significant overestimation of 97.5 mm (11.4%). The discrepancy was most pronounced in August (39.5 mm) and July (27.3 mm), with smaller but agronomically relevant overestimations across all remaining growing-season months. From an agronomic management perspective, relying on TSMS data for irrigation scheduling would lead to an over-application of approximately 97.5 m^3^ of water per decare over the growing period. To illustrate the practical magnitude of this inefficiency, consider a representative commercial vineyard of 50 da, a scale typical of mid-sized family operations in the Antalya region [40], for which the cumulative excess water application would reach approximately 4875 m^3^ per season (calculated as 97.5 m^3^/da × 50 da). Such a surplus represents a critical inefficiency in water resource management, particularly within the Mediterranean region, where increasing water scarcity necessitates highly precise irrigation protocols to maintain sustainable production.

## 4. Discussion

### 4.1. Magnitude and Significance of ET_0_ Deviations

It should be noted that the TSMS station serves as a standardized regional benchmark in this study rather than an absolute meteorological ground truth. Given the contrasting surface exposure conditions between the two sites—an actively transpiring vineyard canopy versus an urban setting—systematic differences in wind speed, relative humidity, and net radiation are physically expected and do not reflect instrument error at either station. The comparative analysis presented herein therefore quantifies the agronomic consequences of spatial data mismatch rather than the accuracy of either measurement system in absolute terms.

The findings of this study provide quantitative evidence that regional meteorological data, as represented by TSMS records, diverges substantially from field-level measurements for ET_0_ estimation. The paired *t*-test (t = −3.51, *p* = 0.005) confirms a statistically significant mean difference between the two data sources, rejecting the hypothesis of interchangeability. The full suite of error metrics is reported in Table 5 and discussed in Section 3.4. Acknowledging that these results are derived from a single calendar year (2021) and should be interpreted within that temporal scope, these findings are nonetheless consistent with those of Allen et al. [4], who emphasized the fundamental dependence of Penman–Monteith ET_0_ accuracy on the spatial representativeness of meteorological inputs, and corroborate the substantial inter-station ET_0_ deviations documented by Gavilán et al. [61] and Vanderlinden et al. [62] across semiarid Mediterranean environments.

The statistical significance of the discrepancy (*p* = 0.005) is driven by a temperature-dependent intensification of TSMS overestimation across both seasons: the cool-season mean bias (MBE = −0.49 mm/day; October–March) and the warm-season mean bias (MBE = −0.91 mm/day; April–September) together yield an annual mean MBE of −0.695 mm/day—indicating that TSMS consistently overestimates field-level ET_0_ throughout the year, with the magnitude of overestimation increasing by approximately 86% from the cool to the warm season (from 0.49 to 0.91 mm/day). This differential bias magnitude between seasons underscores why aggregate statistics alone may mask the temporally heterogeneous nature of the overestimation, making them insufficient for evaluating the suitability of regional data for ET_0_ estimation; complementary error magnitude metrics (RMSE, MAE) and model efficiency indices (NSE, Willmott’s d) are essential for a complete assessment of data source reliability in precision irrigation applications.

### 4.2. Seasonal Dynamics and Multi-Pathway Temperature-Driven Bias

The significant inverse correlation between mean monthly air temperature and ET_0_ deviation (ΔET_0_ = −0.071T + 0.747; r = −0.67, R^2^ = 0.45, *p* = 0.016, n = 12) confirms a systematic, temperature-indexed bias structure. A variable-by-variable attribution analysis (Table 6) reveals that this bias is transmitted through three primary physical pathways, each operating simultaneously and amplifying with seasonal temperature. First, wind speed differences account for the largest individual contribution to the ET_0_ discrepancy (approximately 36.6% of explainable ΔET_0_; mean Δu_2_ = −1.08 m/s). The vineyard canopy provides consistent aerodynamic sheltering throughout the year, maintaining lower wind speeds at the field station relative to the exposed, urban TSMS site. This sheltering effect is particularly consequential in the Penman–Monteith aerodynamic term, which scales with both u_2_ and vapor pressure deficit (VPD): at higher VPD—which itself intensifies with temperature—the aerodynamic term amplifies the wind speed difference into a progressively larger ET_0_ contribution. Second, relative humidity differences contribute approximately 30.0% of the discrepancy (mean ΔRH = +5.6%). The vineyard canopy sustains evaporative cooling and transpirational moisture release, maintaining systematically higher relative humidity than the paved, unvegetated TSMS environment throughout the year. During peak summer months, this divergence reaches 13 percentage points (July: RH_Field_ = 59%, RH_TSMS = 46%), substantially reducing the vapor pressure deficit at the field station and thereby lowering ET_0_ relative to the TSMS estimate. Third, net radiation differences contribute approximately 31.0% of the discrepancy, particularly during cool-season months when ΔRn between sites is more variable. Although both stations experience broadly comparable incoming shortwave radiation loads, differences in surface albedo, longwave emission, and land-surface energy partitioning between the vegetated vineyard and the urban TSMS environment produce measurable Rn divergences at certain periods of the year. The direct contribution of air temperature differences (ΔT) to the ET_0_ discrepancy was modest (approximately 2.4%), reflecting the small annual mean temperature difference between the two sites (−0.09 °C). However, temperature operates as a higher-order driver whose influence is transmitted indirectly: elevated temperatures at the TSMS site—driven by the urban heat island effect [57,58]—coincide with reduced relative humidity, unchanged or elevated wind exposure, and altered surface energy partitioning. This multi-pathway structure explains why T_Field_ serves as an effective seasonal index for ΔET_0_ in the regression model, despite its modest direct contribution to the energy balance discrepancy: when T_Field_ is high, the full suite of microclimate differences between the vineyard and the urban station simultaneously intensifies, producing the negative T–ΔET_0_ relationship observed. Notably, the identified contributors—wind speed attenuation, net radiation differences, and relative humidity divergence—are direct physical consequences of vineyard canopy structure and the contrasting exposure characteristics of the two stations, rather than artefacts of site-specific topography; their consistent expression and coherent seasonal amplification across all twelve months support this interpretation. The attribution percentages in Table 6 should nonetheless be interpreted as structured approximations of relative variable contribution; as with all one-at-a-time sensitivity approaches, nonlinear interactions among variables are not independently resolved, and the approximately 19% residual between the sum of individual contributions and the observed annual mean ΔET_0_ reflects the magnitude of these compound effects—a finding that itself motivates future variance decomposition analyses. Similar multi-variable spatial discrepancies in ET_0_ estimates between regional stations and local agricultural measurements have been documented across Mediterranean environments [61,62], though comprehensive attribution analyses of this type remain rare in the literature, limiting direct quantitative comparison. It should be noted that the one-at-a-time (OAT) substitution approach employed in Table 6 cannot independently resolve the nonlinear interactions inherent in the Penman–Monteith equation. The 19% residual between the sum of individual contributions (0.563 mm/day) and the observed annual mean ΔET_0_ (0.695 mm/day) confirms that compound interactions are non-negligible. The reported percentages should therefore be interpreted as structured first-order approximations rather than precise causal decompositions. Methods such as variance decomposition or Shapley value-based attribution are recommended in future work to fully resolve the nonlinear contribution structure.

### 4.3. Practical Implications for Irrigation Management

The irrigation scenario analysis confirms that the agronomic consequences of ET_0_ deviation are substantial. As detailed in Section 3.6, reliance on TSMS data leads to an 11.4% seasonal overestimation of ET_C_ (97.5 mm), primarily concentrated in July and August. As detailed in Section 3.6, this surplus represents a critical inefficiency under the water-scarce conditions characteristic of the Mediterranean region.

Equally important is the temporal pattern of overestimation. During the early growing season (April–May), TSMS data overestimates ET_C_ by a combined 5.8 mm (April: 2.6 mm; May: 3.2 mm). Although smaller in absolute magnitude than the peak-season discrepancy, this early-season overestimation may promote unnecessary water application during budbreak and the initial vegetative stages, with potential consequences for soil aeration, nutrient leaching, and fungal disease pressure. Budbreak and flowering are particularly sensitive phenological windows during which irrigation management decisions can irreversibly affect yield potential and berry quality [41,42]. Overestimation during peak summer months not only generates unnecessary water expenditure but may also promote waterlogging, accelerate nutrient leaching, and elevate disease pressure through excessive canopy humidity. In total, TSMS-based overestimation persists across the entire growing season, with the magnitude escalating consistently with rising temperature—underscoring that the challenge lies not merely in the total seasonal water quantity, but in the temporal precision of irrigation scheduling, a dimension that regional data sources are structurally unable to provide.

### 4.4. System Capabilities and Operational Advantages

In addition to the ET_0_ and ETc discrepancies quantified above, the monitoring system developed in this study demonstrates the feasibility of deploying a low-cost, modular Internet of Farming (IoF) architecture in commercial vineyard environments. The named hardware components—including the sensor suite, data acquisition (DAQ) hardware, communication module, and power supply—totaled US$851.69. Including the enclosure, mounting mast, cabling, connectors, and RS485 interface hardware, the per-unit system cost was US$970–1050 (Supplementary Materials, Table S3). This cost is substantially lower than the publicly available list prices of commercial precision agriculture monitoring systems with comparable environmental monitoring capabilities, which range from US$1756 to US$3735 (Supplementary Materials, Table S4). Beyond its economic advantage, the proposed system simultaneously acquires atmospheric, soil, and irrigation water quality data, thereby providing a more comprehensive decision-support platform than ET_0_ estimation alone. In particular, the integration of irrigation water pH and electrical conductivity (EC) monitoring addresses an important aspect of irrigation management that is not provided by regional meteorological services but is fundamental to maintaining soil health, optimizing nutrient management, and supporting sustainable vineyard production.

The modular architecture and cloud-based data infrastructure also offer considerable scalability. This scalability is rooted in three specific architectural features: the modular One-Wire/I^2^C sensor bus (Section 2.2) allows additional sensing modules to be incorporated into an existing field station without redesigning the acquisition electronics; reliance on standard cellular (GSM/GPRS) connectivity permits new stations to be deployed wherever cellular coverage exists, independent of site-specific network infrastructure; and the cloud application layer’s role-based, multi-tenant design (Figure 5) allows data from multiple field stations to be managed under a single administrative account. In future deployments, a spatially distributed network of field stations across a production region could capture intra-regional microclimatic variability, thereby enabling variable-rate irrigation prescriptions tailored to sub-field conditions. Furthermore, the real-time threshold alarm system—capable of detecting frost events, heat stress episodes, and anomalous pH or EC values—adds a proactive risk management capability that cannot be provided by regional meteorological services at the field scale. This combination of continuous monitoring, remote accessibility, and automated alerting positions the proposed IoF platform as a versatile framework with promising applicability beyond viticulture. Nevertheless, further multi-site and multi-year evaluations across diverse crops and pedoclimatic conditions are required to validate its broader applicability.

### 4.5. Limitations and Future Research

Several limitations of this study warrant acknowledgment. First, the reliance on monthly mean ET_0_ results in a constrained inferential sample size (n = 12). While this temporal aggregation aligns with standard crop coefficient-based irrigation scheduling and seasonal ET_C_ frameworks, results should be interpreted in the context of this temporal scale; daily resolution analyses in future studies would provide a complementary perspective on the short-term dynamics of ET_0_ discrepancies. Second, the study’s scope is restricted to a single vineyard site during a single calendar year (2021). While site-specific topographic effects cannot be entirely excluded, the variable-by-variable attribution analysis (Table 6) identifies wind speed attenuation, relative humidity divergence, and net radiation differences—all direct consequences of vineyard canopy structure and station exposure characteristics—as the dominant physical drivers of the observed bias, which argues against a topographically artefactual origin. Regarding interannual representativeness, the near-monotonic directional consistency of the bias across 11 of 12 months, with a seasonal structure that intensifies systematically with temperature rather than fluctuating irregularly, is more consistent with the operation of persistent, structurally determined drivers than with a transient climatic anomaly specific to 2021. Nonetheless, multi-year validation across diverse pedoclimatic settings remains essential to confirm the stability of the identified temperature–ET_0_ relationship across years with contrasting thermal and moisture regimes, and this is explicitly identified as a priority direction for future research. Third, the use of static FAO-56 Kc values in this study was a deliberate methodological choice to isolate ET_0_-driven discrepancies from Kc-related uncertainty in the comparative analysis between the IoF field station and TSMS data sources. Nevertheless, it is acknowledged that the IoF architecture deployed here simultaneously acquires soil volumetric water content (VWC) and time-series canopy imagery—data streams that could, in future work, provide the observational basis for empirical Kc adjustment approaches, such as FAO-56 dual-coefficient methods or NDVI-based Kc derivation frameworks. However, realising this integration requires sufficient accumulation of phenological observations and site-specific calibration, prerequisites that lie beyond the scope of the present study. The need for more accurate, dynamically updated Kc estimates—informed by real-time canopy state and soil moisture data—represents a meaningful limitation of the current irrigation decision-support framework and is identified as a priority direction for subsequent system development. Fourth, the estimation of saturation vapor pressure (e_s_) from mean daily temperature, rather than the FAO-56 recommended (T_max_/T_min_) averaging, may lead to a marginal underestimation of ET_0_, particularly during periods of high diurnal temperature range. Fifth, although the IoF monitoring station operated continuously under field conditions for one year, periodic laboratory recalibration of the sensors was beyond the scope of this study. Therefore, the potential long-term sensor drift resulting from prolonged outdoor exposure, photovoltaic-powered operation, and cyclic temperature and humidity variations was not quantitatively evaluated. Consequently, the cumulative uncertainty that sensor drift may introduce into long-term ET_0_ estimation could not be quantified from the available dataset. Future studies should incorporate scheduled sensor recalibration and long-term stability assessments to characterize drift-induced uncertainty under continuous field operation. Sixth, although the proposed IoF monitoring station was powered by a standalone photovoltaic system to enable uninterrupted autonomous field operation, the electrical power consumption of the individual hardware components and the overall station energy budget were not quantified because energy performance evaluation was beyond the scope of the present study. Future research will include detailed power consumption measurements and energy budget analyses under different environmental and operating conditions to further improve the long-term energy efficiency and sustainability of the proposed IoF monitoring system. Finally, the attribution analysis employs a one-at-a-time substitution approach, which does not independently resolve nonlinear variable interactions; the directional conclusions regarding the dominant physical drivers remain robust, but the reported percentages should be interpreted as structured approximations rather than precise causal decompositions, as further evidenced by the 19% residual between the sum of individual contributions and the observed ΔET_0_. Future research should therefore employ more rigorous partitioning methods, such as variance decomposition.

Notwithstanding these limitations, the findings presented here provide a robust initial framework for quantifying microclimate-driven ET_0_ deviations and their irrigation scheduling consequences, with clear directions for future validation and system development.

Future research should prioritize the following areas:

- Correction Modeling: Developing and validating temperature-indexed empirical correction functions across multiple sites and years, to evaluate whether the bias relationship identified in the present study can be generalized for operational application in regions lacking localized monitoring infrastructure.
- Spatial Variability: Deploying multi-station sensor networks to quantify intra-field and inter-field microclimatic heterogeneity and enable variable-rate irrigation prescriptions.
- Closed-Loop Automation: Integrating the IoF monitoring platform with automated irrigation controllers and decision algorithms to achieve feedback-driven, real-time precision irrigation management, thereby eliminating dependence on manual scheduling based on static reference data.
- Long-term Climate Resilience: Leveraging longitudinal, multi-year field datasets to assess interannual variability in ET_0_ deviations and develop predictive models for climate adaptation and agricultural risk management.

## 5. Conclusions

This study developed and field-deployed a low-cost, modular IoF monitoring system in a commercial vineyard to systematically quantify the discrepancies between localized field-level and regional TSMS-derived ET_0_ estimates, and to evaluate their implications for grapevine irrigation management. The principal conclusions are as follows:

- Data Divergence: Official TSMS data and localized field station measurements yield significantly different ET_0_ estimates (paired *t*-test: t = −3.51, *p* = 0.005), characterized by an RMSE of 0.956 mm/day, an MAE of 0.701 mm/day, and a MAPE of 10.56% as presented in Table 5. The NSE of 0.8466 confirms very good predictive correspondence between the two sources, while Willmott’s d of 0.9679 and R^2^ of 0.963 reflect strong seasonal synchronization—together demonstrating that the discrepancy manifests as a systematic, multi-pathway, temperature-indexed overestimation by TSMS rather than random disagreement. The comparable seasonal variability of both data sources (CV_TSMS_ = 50.26%; CV_Field_ = 48.66%) confirms that the systematic bias rather than differential temporal dispersion is the dominant source of irrigation scheduling error.
- Temperature-Indexed Multi-Pathway Bias: ET_0_ deviation exhibits a systematic, temperature-indexed intensification of TSMS overestimation, characterized by the monthly regression ΔET_0_ = −0.071T + 0.747 (r = −0.67, R^2^ = 0.45, *p* = 0.016, n = 12). A variable-by-variable attribution analysis (Table 6) identifies wind speed differences (Δu_2_; ~36.6%), net radiation differences (ΔRn; ~31.0%), and relative humidity differences (ΔRH; ~30.0%) as the primary physical contributors to ET_0_ discrepancy, while the direct contribution of air temperature differences was modest (~2.4%), suggesting that field station air temperature functions primarily as a seasonal index variable for the integrated microclimate divergence rather than as a direct causal driver—a relationship warranting confirmation across additional sites and years.
- Irrigation Inefficiency: Reliance on regional TSMS data for grapevine irrigation scheduling results in an 11.4% overestimation of the seasonal crop water requirement (ET_C_), corresponding to 97.5 mm (97.5 m^3^/da) of excess water application over the growing season. For a representative commercial vineyard of 50 da [40], this amounts to approximately 4875 m^3^ of unnecessary water use per season (calculated as 97.5 m^3^/da × 50 da).
- Decision Support Potential: The IoF-based monitoring system provides real-time, field-level environmental data that substantially enhances the precision and reliability of irrigation decision-making. The identified temperature–ET_0_ deviation relationship offers a physically grounded basis for developing empirical correction functions in regions where localized monitoring infrastructure is unavailable; translating this relationship into operational tools represents a productive avenue for future multi-site, multi-year research.

Collectively, these findings provide quantitative evidence for the significant agronomic and resource-conservation benefits of site-specific IoF monitoring for precision agriculture in Mediterranean viticulture—within the specific site and year examined. The broader applicability of the identified temperature-indexed correction framework across other crops, climate regimes, and management systems represents a promising research avenue that the present findings motivate and inform, but that future multi-site, multi-year investigations are necessary to establish. The temporal precision and field-level accuracy afforded by localized sensing are particularly vital in Mediterranean viticulture, where escalating water scarcity, increasing climate variability, and the economic sensitivity of grape production demand increasingly reliable and spatially representative decision-support systems.

## Figures and Tables

**Table 1:** Comparison of representative low-cost agricultural IoT sensing platforms with the proposed IoF framework.

Study	Platform	Communication	Power Supply	Microclimate Monitoring	ET_0_ Estimation	Reference Data Comparison *	Primary Application
Jimenez-Berni et al. (2023) [31]	Wireless Sensor Network	ZigBee	Solar	✓	✓	✗	Crop ET monitoring
Smart Reference ET (2023) [32]	IoT sensor nodes	IoT wireless network	Battery/Grid	✓	✓	✓	Smart irrigation
Microclimate Monitoring System (2023) [33]	IoT sensor nodes	Wi-Fi	Solar	✓	✗	✗	Irrigation water optimization
Hasan et al. (2025) [34]	ESP32-based sensor node	Wi-Fi	Solar	✓	✗	✗	Smart fertigation
Chandel et al. (2025) [35]	UAV + thermal/spectral sensors	Wireless	Battery	✓	✓	✓	Orchard water management
This study	ESP32 + Raspberry Pi	Wi-Fi	Standalone solar PV	✓	✓	✓	Vineyard irrigation management

* Reference Data Comparison indicates whether the proposed measurements or ET_0_ estimates were evaluated against independent reference meteorological observations, benchmark datasets, or reference ET calculations reported in the respective studies. (“✓” indicates that the feature is included/supported, whereas “✗” indicates that it is not included/reported in the respective study).

**Table 2:** Technical specifications, measurement ranges, accuracy levels, and resolution of the sensors integrated into the IoF field station.

Measurement	Sensor Type	Measuring Range	Accuracy	Resolution
Air temperature	Polymer capacitor	−40–60 °C	±0.5 °C	0.1 °C
Relative humidity	Polymer capacitor	0–100% rh	±2% rh	0.1 rh
Barometric pressure	Digital air pressure	300–1100 hPa	±1 hPa	0.03 hPa
Wind speed	Hall effect sensor	0.9–35 m/s	±2%	0.1 m/s
Wind direction	Weathervane	0–360°; 16 points	±3°	1°
Rainfall	Pulse counter	0–999.8 mm	0.2 mm	0.2 mm
Solar radiation	Silicon-cell (350–1100 nm)	0–2000 W/m^2^	±5%	1 W/m^2^
Leaf wetness	Artificial leaf	0–15 scale	±0.5	1
Soil temperature	S-Temp.; VWC; EC-02A	−40–80 °C	±0.5 °C	0.1 °C
Soil moisture	S-Temp.; VWC; EC-02A	0–100%	±3% (0–50%)	0.03%
Electrical conductivity	S-Temp.; VWC; EC-02A	0–10,000 μs/cm	±3%	10 μs/cm
pH	Gel compact electrode	0–14	±0.1	0.01

**Table 3:** Monthly mean values of meteorological and thermodynamic variables measured at the field station and used as inputs for FAO-56 Penman–Monteith ET_0_ calculations during the 2021 monitoring period.

Parameters	Months											
	Jan	Feb	Mar	Apr	May	Jun	Jul	Aug	Sep	Oct	Nov	Dec
T_Field_ (°C)	12.2	12.6	14.3	17.1	22.8	26.8	29.7	28.9	26.8	20.2	18.1	14.1
T_TSMS_ (°C)	11.6	12.6	13.0	17.1	22.9	25.5	31.3	31.2	26.1	21.7	18.5	13.2
RH_FIELD_ (%)	70	68	69	73	70	66	59	60	66	69	69	75
RH_TSMS_ (%)	77	69	63	68	64	61	46	48	57	53	67	74
u_2FIELD_ (m/s)	1.5	1.7	1.8	1.4	1.3	1.4	1.2	1.2	1.4	1.5	1.4	1.5
u_2TSMS_ (m/s)	2.2	2.5	2.7	2.6	2.6	2.7	2.8	2.7	2.7	2.2	2.1	2.5
γ_FIELD_ (kPa/°C)	0.0670	0.0670	0.0670	0.0670	0.0670	0.0670	0.0670	0.0670	0.0670	0.0670	0.0670	0.0670
γ_TSMS_ (kPa/°C)	0.0670	0.0670	0.0670	0.0670	0.0670	0.0670	0.0670	0.0670	0.0670	0.0670	0.0670	0.0670
R_n,FIELD_ (MJ/m^2^·d)	7.60	10.63	14.50	20.78	23.35	25.84	29.20	21.79	20.20	14.52	9.67	7.11
R_n,TSMS_ (MJ/m^2^·d)	8.09	11.68	16.90	21.47	22.64	28.40	27.73	23.97	19.10	16.59	11.00	6.77
Δ_FIELD_ (kPa/°C)	0.0936	0.0957	0.1055	0.1235	0.1681	0.2070	0.2397	0.2303	0.2070	0.1463	0.1305	0.1043
Δ_TSMS_ (kPa/°C)	0.0904	0.0957	0.0980	0.1235	0.1690	0.1936	0.2596	0.2583	0.1997	0.1586	0.1334	0.0991
es_FIELD_ (kPa)	1.4212	1.4590	1.6300	1.9500	2.7756	3.5237	4.1706	3.9826	3.5237	2.3674	2.0770	1.6090
es_TSMS_ (kPa)	1.3660	1.4590	1.4978	1.9500	2.7925	3.2634	4.5699	4.5440	3.3814	2.5960	2.1298	1.5175
ea_FIELD_ (kPa)	0.9948	0.9921	1.1247	1.4235	1.9429	2.3257	2.4607	2.3896	2.3257	1.6335	1.4331	1.2068
ea_TSMS_ (kPa)	1.0518	1.0067	0.9436	1.3260	1.7872	1.9906	2.1022	2.1811	1.9274	1.3759	1.4270	1.1229

**Table 4:** Monthly ET_0_ estimates (mm/day) derived from field station and TSMS data, with absolute difference (Δ = ET_0_,Field − ET_0_,TSMS, mm/day) and relative (Δ%) differences expressed relative to the TSMS estimate for the 2021 calendar year.

Data Source	Months												Mean
	Jan	Feb	Mar	Apr	May	Jun	Jul	Aug	Sep	Oct	Nov	Dec	
Field − ET_0_	2.18	2.89	3.82	5.40	6.88	8.24	9.77	7.48	6.68	4.42	3.06	2.09	5.24
TSMS − ET_0_	2.14	3.16	4.38	5.68	7.09	9.12	11.03	9.80	7.15	5.89	3.64	2.16	5.94
Δ (mm/day)	+0.04	−0.27	−0.56	−0.28	−0.21	−0.88	−1.26	−2.32	−0.47	−1.47	−0.58	−0.07	−0.695
Δ (%)	1.9	8.6	12.8	5.0	2.9	9.7	11.4	23.7	6.6	25.0	16.0	3.2	10.56

**Table 5:** Statistical metrics evaluating the correspondence between field station and TSMS-based ET_0_ estimates. Pearson r, R^2^, and the paired *t*-test were computed on the monthly means (n = 12); all remaining metrics (RMSE, MAE, MBE, MAPE, NSE, Willmott’s d, CV) were derived from monthly means (n = 12).

Statistical Metric	Value	Interpretation
Paired *t*-test (t)	−3.51	*p* = 0.005 Statistically significant (*p* < 0.01); null hypothesis rejected
Paired *t*-test (p)	0.005	*p* < 0.01; null hypothesis rejected at α = 0.05
Wilcoxon (W)	1.00	W = 1 indicates near-complete rank dominance; consistent directional asymmetry confirmed.
Wilcoxon (p)	0.001	*p* < 0.001; highly significant
RMSE (mm/day)	0.956	Moderate total prediction error magnitude
MAE (mm/day)	0.701	Average absolute error between field and TSMS estimates
MBE (mm/day)	−0.695	TSMS consistently overestimates field-level ET_0_
MAPE (%)	10.56	10.6% mean percentage deviation of TSMS from field-level ET_0_
Pearson r	0.9814	Very strong linear correlation
R^2^	0.9631	96.3% of ET_0_ variance explained; strong seasonal co-variation
NSE	0.8466	Very good model performance (NSE > 0.75); Moriasi et al., [60]
Willmott’s d	0.9679	Near-perfect agreement index
CV–Field (%)	48.66	High seasonal variability
CV–TSMS (%)	50.26	High seasonal variability

**Table 6:** Attribution of ET_0_ discrepancies between the field station and TSMS.

Variable	Annual Mean Contribution (mm/day)	Warm Season (Apr–Sep) (mm/day)	Cool Season (Oct–Mar) (mm/day)	% of Total Explained ΔET_0_	Physical Pathway
ΔT (°C)	+0.013	+0.038	−0.011	2.4%	Minimal direct contribution
ΔRH (%)	+0.169	+0.248	+0.089	30.0%	VPD amplification at TSMS site
Δu_2_ (m/s)	+0.206	+0.278	+0.134	36.6%	Largest contributor; vineyard wind shelter
ΔRn (MJ/m^2^·d)	+0.175	+0.096	+0.253	31.0%	Surface energy partitioning
Total attributable	+0.563	+0.660	+0.465	100%	

Note: Contributions were estimated by replacing field station values with TSMS data in the FAO-56 Penman–Monteith equation, holding other inputs constant. Positive values indicate a contribution to TSMS overestimation. Percentages are normalized to the sum of contributions (0.563 mm/day); the residual reflects nonlinear interactions. The limited impact of ΔRn during the warm season stems from Field recording higher Rn values than TSMS in May, July, and September, thereby offsetting the net seasonal contribution. Abbreviations: ΔT = monthly mean air temperature difference (T_Field_ − T_TSMS_); ΔRH = relative humidity difference (RH_Field_ − RH_TSMS_); Δu_2_ = wind speed difference at 2 m (u_2_,_Field_ − u_2_,_TSMS_); ΔRn = net radiation difference (Rn,_Field_ − Rn,_TSMS_). Positive contributions to ΔET_0_ overestimation indicate that the TSMS value of the respective variable exceeds that of the field station in a direction that increases ET_0_.

**Table 7:** Monthly crop evapotranspiration (ET_C_) estimates for grapevine (*Vitis vinifera* L.) during the 2021 growing season, comparing field station and TSMS-derived values.

Month	K_C_	Number of Day	ET_0_, Field (mm/day)	ET_0_, TSMS (mm/day)	ET_C_, Field (mm/month)	ET_C_, TSMS (mm/month)	ΔET_C_ (mm/month)	Δ%
Apr	0.30	30	5.40	5.68	48.5	51.1	−2.6	5.0
May	0.50	31	6.88	7.09	106.7	109.9	−3.2	2.9
Jun	0.70	30	8.24	9.12	173.0	191.5	−18.5	9.7
Jul	0.70	31	9.77	11.03	212.1	239.4	−27.3	11.4
Aug	0.55	31	7.48	9.80	127.6	167.1	−39.5	23.7
Sep	0.45	30	6.68	7.15	90.1	96.5	−6.4	6.6
Total		183			758.0	855.5	−97.5	11.4

## Data Availability

The original contributions presented in the study are included in the article. Further inquiries can be directed at the corresponding author.

## Supplementary Materials

The following supporting information can be downloaded at: https://www.mdpi.com/article/10.3390/s26165154/s1, Figure S1: Additional panels of the cloud-based IoF monitoring dashboard: (a) soil temperature (°C) and (b) global solar radiation (W/m^2^) time-series records acquired during the 2021 monitoring period; Table S1: Temperature values; Table S2: Data Collected from the IoF Field Monitoring Station; Table S3: Bill of Materials and Unit Cost of the Proposed IoF Field Monitoring Station; Table S4: Publicly Available Market Prices of Commercial Precision Agriculture Monitoring Systems [63,64,65].
