# Supplementary material for: Development and Validation of an IoF-Based Sensing Architecture for Microclimate Monitoring: Quantifying Spatial ET0 Bias in Mediterranean Viticulture
Source: Sensors (Basel). 2026 Aug 14;26(16):5154. doi: 10.3390/s26165154 (PMC13517316; doi:10.3390/s26165154)
Supplement: Supplementary file 1 [file sensors-26-05154-s001.zip › sensors-4405419-supplementary.pdf]

Supplementary Materyals

1. Figures

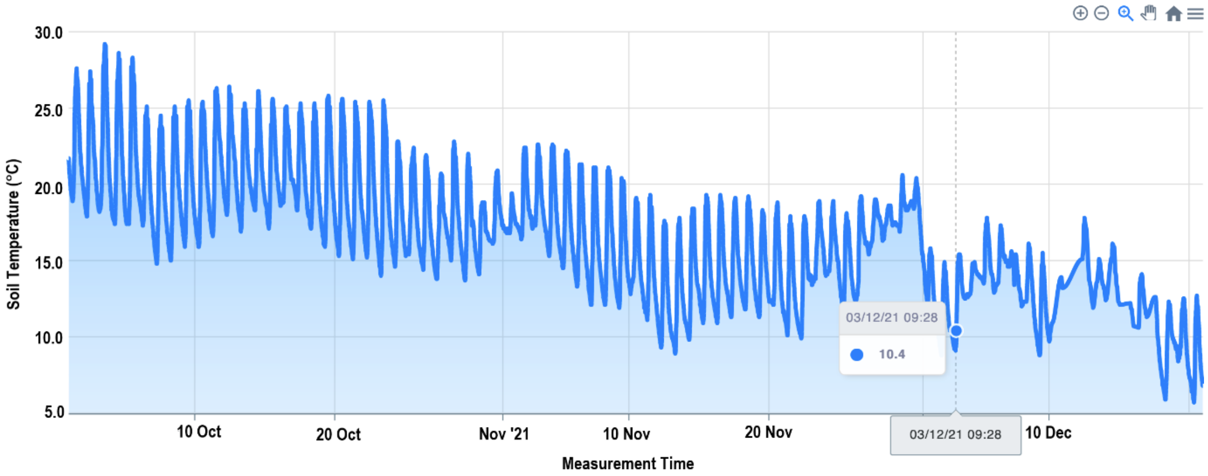

(a)

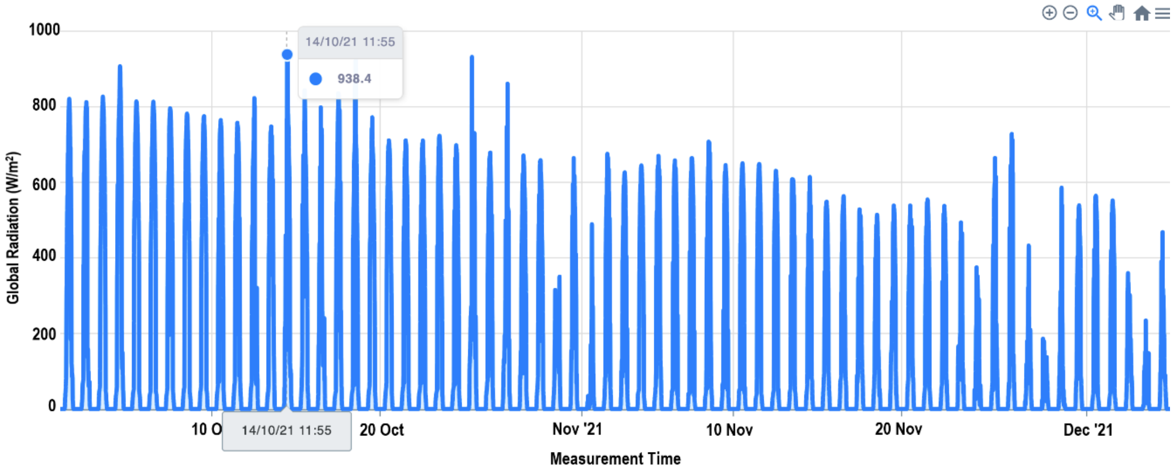

(b)

Figure S1. Additional panels of the cloud-based IoF monitoring dashboard: **(a)** soil temperature (°C) and **(b)** global solar radiation (W/m²) time-series records acquired during the 2021 monitoring period.

2. Tables

Table S1. Temperature values.

Gün/Ay	Jan	Feb	Mar	Apr	May	Jun	Jul	Aug	Sep	Oct	Nov	Dec	Mean
1	13.2	9.3	14.2	11.5	24.2	21.2	34.8	36	27.8	25.5	17.3	15.7	20.9
2	13.6	16.4	11.3	12.7	23.7	21.8	29.3	35.7	30.8	23.2	21	13.8	21.1
3	12.5	14.7	12.2	13.7	22.9	23.5	28.3	36.6	30	25.7	19.9	13.2	21.1
4	12.4	14	14.9	15.7	21.9	24.6	31.2	36	27.8	24.6	20.2	13.6	21.4
5	12.1	14.7	12.1	15.8	22.9	25.4	30	33.7	27	24.2	19	17.8	21.2
6	13.9	13	14	16.2	21.5	25.7	28.3	30.3	26.7	23.6	18.6	15.7	20.6
7	14	12.3	13.4	16.9	24.3	23	30.1	29.3	27.8	21.2	20.7	17	20.8
8	13.8	13.5	15	16.1	21.4	22	31	30.8	27.2	21.7	19.8	14.6	20.6
9	13.9	16.1	12.7	15.4	25.8	22.8	32.7	32.8	26.9	21.8	18.9	12.8	21.1
10	15.4	14.8	14	12.7	25.7	22.2	33.1	33.5	27.3	21.5	19.8	13.8	21.2
11	14	13.5	14	13	21.2	22.4	32.1	34.7	27.7	23.4	20.1	15.3	21.0
12	14.7	13.7	14.2	14.3	20.3	22.2	30.2	32.8	26.1	22.2	15.9	19.2	20.5
13	17.6	14.7	12.3	13.7	20.5	22.8	29.2	32.3	25.3	22.3	17.6	14.2	20.2
14	13.2	12.2	12.7	14.3	20.7	22.3	31	32.6	25	21.8	17.5	14.6	19.8
15	10.1	12	12.6	15.9	20.2	22.5	33.7	31.8	21.9	21.6	19.2	12.9	19.5
16	10.7	7.9	14.5	15.9	19.8	21.5	34.2	31.7	25.8	21.1	18.5	12.7	19.5
17	11.2	6.1	12.1	18	22.3	24.4	30.2	29.7	27	22	18.2	11.7	19.4
18	7.2	8.1	12.4	21.3	21.3	25	28	27.8	25.4	22.2	18.2	10.9	19.0
19	8.1	10.4	11.5	21.6	22.5	24.4	28.4	28.1	25.9	21.6	17.1	9.7	19.1
20	7.4	9	13.6	19.7	21.5	23.5	27	30.5	26.3	21.1	17.5	12.3	19.1
21	7.2	9.6	14	17.1	21	23.6	27.2	30.5	26.8	20	17.5	10.9	18.8
22	8.5	9.4	15.8	16.8	23.3	23.6	28.2	29.9	29.7	20	16.2	9.2	19.2
23	9.7	11.6	16.3	17.1	26.1	26.3	31.1	31.6	26.2	19.9	16.5	8.3	20.1
24	10.7	17.5	12.6	19.6	27.5	29.2	31.1	31.4	22.7	19.2	16.1	9.9	20.6
25	11.3	17	8.7	21.1	29.5	31.9	30.7	29.6	22.8	21.7	17	10.8	21.0
26	12.5	14.6	10	23.2	24.9	32.7	32.9	29	22.6	20.7	16.3	12	21.0
27	13.9	12.7	12.2	21.7	21.9	34	33.2	28	23.7	19.6	18.9	13.4	21.1
28	8.9	13	11.5	18.8	22.1	33.6	35.3	27	25.3	21.5	19.3	13	20.8
29	8.9		12.5	19.9	24.4	34.1	35.6	27.3	22.9	19.4	19.7	12.2	21.5
30	5.6		13.4	21.9	22.8	34.2	35.7	27.8	24.7	18.7	21.1	14.3	21.8
31	12.4		13.7		22.8		36	27.8		19.1		13	20.7
Mean	11.6	12.6	13.0	17.1	22.9	25.5	31.3	31.2	26.1	21.7	18.5	13.2	

Table S2. Data Collected from the IoF Field Monitoring Station

Date anad time	Irrigation water temperature (°C)	Irrigation water, EC (mS/cm)	Irrigation water, pH	Air temperature (°C)	Air humidity (%)	Rainfall (mm)	Barometric pressure (mbar)	Wind direction	Wind velocity (km/h)	Soil moisture (%)	Soil temperature (°C)	Solar radiation (W/m^2)
2021-09-23 08:07:28	20.9	0.8	6.2	25.9	11	0	1001.1	S	7.2	21	21.5	223
2021-09-23 08:22:57	18.7	0.9	5.8	26	11	0	1001.1	S	8.7	21	21.7	294
2021-09-23 08:38:25	19.1	0.9	6.0	26.2	11	0	1002	SE	10.3	21	22	156
2021-09-23 08:53:55	19.3	0.9	6.2	24.4	12	0	1002.6	W	9.4	22	21.9	84
2021-09-23 09:09:22	20.3	0.9	5.9	24.7	13	0	1002.6	S	10.2	21	22.2	144
2021-09-23 09:24:52	20.8	0.9	6.2	26.1	13	0	1002.1	SE	9.3	21	22.8	306
2021-09-23 09:40:20	21.5	0.9	6.4	25.8	12	0	1002.3	SE	9.6	21	23	168
2021-09-23 09:55:49	21.6	0.9	6.4	27.9	14	0	1001.9	SE	9.1	20	23.5	615
2021-09-23 10:11:16	22.2	0.9	6.4	28.6	14	0	1002.1	S	10.5	20	24.2	726
2021-09-23 10:26:45	23.1	0.9	6.3	28.5	16	0	1002.1	S	9.3	20	24.8	735
2021-09-23 10:42:12	24	0.9	6.3	29	16	0	1001.8	SE	8.9	19	26.4	955
2021-09-23 10:57:42	24.5	0.9	6.8	28.7	17	0	1001.8	W	9.6	20	27.4	965
2021-09-23 11:13:11	24.6	0.9	6.7	26.8	17	0	1002.1	SE	9.2	18	26.9	390
2021-09-23 11:28:40	24.7	0.9	6.6	27.9	18	0	1002.1	NE	9.6	19	27.7	430
2021-09-23 13:30:03	28.5	0.9	6.4	29.1	20	0	1000.8	SE	9.9	17	28.4	741
2021-09-23 13:45:36	28.8	0.9	6.6	32.1	24	0	1000	SE	10.4	20	29.1	924
2021-09-23 14:01:17	29.6	0.9	6.6	32	28	0	999.9	SE	10.8	25	29.3	885
2021-09-23 14:16:51	29.7	0.9	6.5	32.1	28	0	999.9	W	11.5	30	29.5	885
2021-09-23 14:50:58	29	0.9	6.7	30.1	29	0	999.7	SE	11.1	34	30.4	783
2021-09-23 15:06:33	28.7	0.9	6.5	30.9	28	0	999.7	SE	12.7	43	31.4	732
2021-09-23 15:22:01	28.3	0.9	6.9	28.5	30	0	999.5	NW	12.3	42	30.4	596
2021-09-23 15:37:31	28.2	0.9	6.7	31.9	30	0	999.7	SE	12.2	44	30.9	696
2021-09-23 15:53:00	28.4	0.9	7.0	31	31	0	999.9	W	11.3	42	30.5	678
2021-09-23 16:08:30	28.2	0.9	7.1	29.7	32	0	1000	SE	11.7	45	30.3	448
2021-09-23 16:24:00	28	0.9	6.8	29.6	34	0	1000	SE	11.9	45	29.6	556
2021-09-23 16:39:29	27.6	0.9	6.9	29.5	35	0	1000.1	SE	11.5	45	29.1	455
2021-09-23 16:54:59	27.1	0.9	6.7	27.6	35	0	1001.5	NW	12.1	44	28.2	284
2021-09-23 17:10:32	26.5	0.9	6.7	27.1	35	0	1001.7	SE	12.3	41	27.5	287
2021-09-23 17:25:58	26.3	0.9	6.8	28.3	37	0	1000.9	SE	12.8	40	27.1	284
2021-09-23 17:41:26	26	0.9	6.6	26	38	0	1001.9	SE	11.3	41	26.6	97
2021-09-23 18:12:32	24.7	0.9	6.6	24.9	40	0	1002.4	S	10.2	39	25.4	28
2021-09-23 18:28:02	24.1	0.9	6.3	24.4	42	0	1002.8	SE	10.5	37	24.9	19

Table S2. (Devamı)

2021-09-23 18:43:31	23.6	0.9	6.3	23.9	45	0	1003	S	9.2	37	24.5	10
2021-09-23 18:59:00	23	0.9	6.3	23.6	45	0	1003.2	SW	10.1	36	24.1	3
2021-09-23 19:14:32	22.5	0.9	6.3	23.4	48	0	1003.4	SE	9.4	38	23.8	1
2021-09-23 19:30:01	22.1	0.9	6.2	23.2	34	0	1003.5	SE	44.5	51	23.4	1
2021-09-23 19:45:30	21.7	0.9	6.3	22.9	34	0	1003.8	S	40.2	49	23.2	1
2021-09-23 20:01:02	21.3	0.9	6.2	22.8	34	0	1004	SE	42.7	50	22.9	1
2021-09-23 20:16:31	20.9	0.9	6.2	22.6	34	0	1004.3	SE	41.0	50	22.7	1
2021-09-23 20:32:00	20.6	0.9	6.2	22.5	34	0	1004.6	S	42.2	49	22.4	1
2021-09-23 20:47:31	20.3	0.9	6.2	22.4	34	0	1004.9	SE	38.2	49	22.3	1
2021-09-23 22:30:41	18.5	0.9	6.0	20.6	40	0	1006.1	SE	27.8	49	21.1	1
2021-09-23 22:46:11	18.2	0.9	6.0	20.6	39	0	1006.4	SE	27.8	49	20.9	1
2021-09-23 23:01:42	18	0.9	6.2	20.3	40	0	1006.4	SE	27.9	47	20.8	1
2021-09-23 23:17:20	17.8	0.9	6.4	20.4	40	0	1006.2	S	27.4	47	20.6	1
2021-09-24 00:09:54	17.1	0.9	6.3	20.1	38	0	1006.6	SE	25.9	49	20.1	1
2021-09-24 00:25:23	16.9	0.9	6.3	19.7	40	0	1006.6	S	26.6	49	19.9	1
2021-09-24 00:40:51	16.7	0.9	6.3	19.6	39	0	1006.7	SE	23.5	49	19.8	1
2021-09-24 00:56:19	16.6	0.9	6.5	19.5	39	0	1006.8	NE	26.6	49	19.7	0
2021-09-24 01:11:48	16.4	0.9	6.3	19.4	39	0	1006.8	W	22.3	49	19.6	0
2021-09-24 01:27:15	16.2	0.9	5.8	19.1	39	0	1006.6	NE	24.7	49	19.4	0
2021-09-24 01:42:44	16.1	0.9	5.8	19.1	39	0	1006.7	SE	27.9	49	19.3	0
2021-09-24 01:58:12	15.9	0.9	5.8	18.9	40	0	1006.6	SE	29.0	49	19.2	0
2021-09-24 02:13:41	15.8	0.9	6.0	18.7	39	0	1006.8	SE	29.8	49	19.1	0
2021-09-24 02:29:10	15.7	0.9	6.2	18.7	40	0	1006.8	NW	32.2	49	19	0
2021-09-24 02:44:38	15.5	0.9	6.0	18.7	39	0	1006.9	W	31.3	49	18.9	0
2021-09-24 03:00:06	15.4	0.9	6.4	18.4	40	0	1006.8	NE	27.4	49	18.8	0
2021-09-24 03:15:35	15.3	0.9	6.2	18.5	39	0	1006.8	S	34.6	49	18.7	0
2021-09-24 03:31:06	15.2	0.9	6.2	18.4	39	0	1006.9	S	34.0	49	18.6	0
2021-09-24 03:46:36	15.1	0.9	6.2	18.3	40	0	1007	S	29.5	49	18.5	0
2021-09-24 04:02:05	15	0.9	6.2	18.4	40	0	1007.1	S	34.6	49	18.4	0
2021-09-24 04:17:34	14.9	0.9	6.2	18.2	40	0	1007.2	SE	32.0	49	18.4	1
2021-09-24 04:33:02	14.8	0.9	6.2	18.1	40	0	1007.5	S	29.3	49	18.3	1
2021-09-24 04:48:30	14.6	0.9	6.2	17.7	41	0	1007.5	SE	26.7	49	18.2	1
2021-09-24 05:04:00	14.5	0.9	6.2	18	41	0	1007.7	S	31.0	49	18.1	2
2021-09-24 05:19:29	14.5	0.9	6.2	17.8	41	0	1007.8	SE	27.7	49	18.1	15
2021-09-24 05:35:01	14.4	0.9	6.2	17.6	41	0	1008	SE	28.9	49	17.9	41
2021-09-24 07:35:39	13.9	0.9	6.2	19.7	37	0	1006.9	SE	10.0	49	17.8	130
2021-09-24 08:08:00	14.2	0.9	6.4	20.6	36	0	1007.2	SE	30.2	49	18.2	242
2021-09-24 08:23:30	14.5	0.9	6.2	20.9	35	0	1007.4	SE	29.6	49	18.4	299
2021-09-24 08:39:00	14.8	0.9	6.2	21.7	33	0	1007.5	SE	28.8	49	18.8	356
2021-09-24 08:54:30	15.2	0.9	6.2	22.1	32	0	1007.6	SE	29.9	49	19.1	412
2021-09-24 09:09:59	15.7	0.9	6.4	22.3	31	0	1007.7	S	31.7	49	19.4	468
2021-09-24 09:25:27	16.4	0.9	6.2	22.9	32	0	1008	SE	32.8	49	19.8	521

Table S2. (Devamı)

2021-09-24 09:40:56	17.1	0.9	6.2	23.8	30	0	1008.1	SE	29.8	49	20.4	73
2021-09-24 09:56:24	17.5	0.9	6.2	23.8	30	0	1008.2	SE	28.8	49	20.7	432
2021-09-24 10:11:53	18.2	0.9	6.4	24.2	29	0	1008.3	SE	30.7	49	21.4	669
2021-09-24 10:27:21	19.3	0.9	6.2	24.3	29	0	1008.4	SE	35.1	49	21.8	711
2021-09-24 10:42:50	20	0.9	6.2	25.3	28	0	1008.5	W	30.6	48	22.7	748
2021-09-24 10:58:18	20.1	0.9	6.2	25.6	28	0	1008.4	S	32.2	48	25.5	783
2021-09-24 11:13:47	20.5	0.9	6.2	25.7	28	0	1008.4	SE	30.3	48	26.9	810
2021-09-24 11:29:14	21.1	0.9	6.2	26.8	27	0	1008.3	SE	29.3	48	27.8	838
2021-09-24 11:44:44	21.6	0.9	6.2	26.9	27	0	1008.1	SE	28.1	48	27.6	859
2021-09-24 12:00:16	22.3	0.9	6.2	27.9	26	0	1007.9	SE	27.2	48	27.9	877
2021-09-24 12:15:45	23.3	0.9	6.0	28.3	25	0	1007.7	E	27.5	48	27.1	897
2021-09-24 12:31:13	24.2	0.9	5.9	29	25	0	1007.5	SE	27.4	48	26.6	903
2021-09-24 12:46:43	25.1	0.9	5.9	30	25	0	1007.2	E	27.4	48	26.4	902
2021-09-24 13:02:12	26.1	0.9	6.0	30.2	25	0	1007	SW	24.0	48	26.5	899
2021-09-24 13:17:41	27.1	0.9	5.9	30.9	24	0	1006.8	NE	24.3	48	28.2	899
2021-09-24 13:33:10	28	0.9	6.2	31	23	0	1006.4	S	22.6	48	28.6	889
2021-09-24 13:48:39	28.7	0.9	6.2	31.6	23	0	1006.1	SE	26.3	48	30.3	876
2021-09-24 14:04:06	29.1	0.9	6.3	31.7	23	0	1005.8	S	24.7	47	31.1	848
2021-09-24 14:19:37	28.8	0.9	6.3	31.6	23	0	1005.8	SW	28.1	48	30.4	821
2021-09-24 15:49:10	27.8	0.9	6.6	29	30	0	1005.8	E	34.7	48	30.3	618
2021-09-24 16:24:52	27.2	0.9	6.6	28.3	33	0	1005.8	NE	38.2	48	29.9	518
2021-09-24 16:40:20	26.6	0.9	6.7	27.5	33	0	1006.1	E	37.7	48	28.6	444
2021-09-24 16:55:49	26	0.9	7.0	25.2	37	0	1007.6	E	36.8	48	27.4	150
2021-09-24 17:11:17	25.4	0.9	6.8	25.1	38	0	1007.6	E	31.9	48	26.6	113
2021-09-24 17:45:23	24.6	0.9	6.4	27.5	35	0	1006.8	NE	29.7	48	25.6	82
2021-09-24 18:00:53	24.1	0.9	6.2	24.7	42	0	1007.7	E	27.3	48	25.1	53
2021-09-24 18:16:20	23.6	0.9	6.2	24.2	44	0	1007.9	E	27.5	48	24.8	22
2021-09-24 18:31:50	23.2	0.9	6.0	23.8	47	0	1008	E	23.4	48	24.4	20
2021-09-24 18:47:19	22.8	0.9	6.0	23.6	47	0	1008.1	SW	24.1	48	24.1	9
2021-09-24 19:02:50	22.4	0.9	5.8	22.9	49	0	1008.2	NE	22.8	48	23.7	2
2021-09-24 19:18:16	22	0.9	5.8	22.9	48	0	1008.4	SW	21.4	48	23.3	1
2021-09-24 19:33:46	21.5	0.9	5.8	21.7	52	0	1008.6	NE	13.2	49	22.9	1
2021-09-24 19:49:15	21	0.9	5.6	20.8	54	0	1008.7	S	10.9	49	22.4	1
2021-09-24 20:04:43	20.5	0.9	5.6	20.9	53	0	1009.1	SE	12.3	49	22	1
2021-09-24 20:20:11	20.1	0.9	5.6	20.2	58	0	1009.3	SE	14.0	49	21.8	1
2021-09-24 20:35:40	19.7	0.9	5.6	19.8	58	0	1009.4	SE	14.9	49	21.5	1
2021-09-24 20:51:08	19.4	0.9	5.6	19.2	58	0	1009.7	NE	15.7	49	21.3	1
2021-09-24 21:06:36	19.1	0.9	5.4	19.6	56	0	1009.9	SE	16.5	49	21.1	1
2021-09-24 21:22:04	18.7	0.9	5.4	19	59	0	1010.1	SE	13.5	49	20.7	1
2021-09-24 21:37:33	18.3	0.9	5.4	19.1	58	0	1010.2	NE	15.8	49	20.4	1
2021-09-24 21:53:03	18	0.9	5.4	19	56	0	1010.3	SE	15.9	49	20.3	1
2021-09-24 22:08:32	17.8	0.9	5.4	18.1	60	0	1010.5	SE	14.4	49	20.1	1

Table S2. (Devamı)

2021-09-24 22:24:03	17.4	0.9	5.4	17.5	60	0	1010.5	NW	14.3	49	19.8	1
2021-09-24 23:49:57	15.8	0.9	5.6	16.5	58	0	1010.9	S	13.5	49	18.6	0
2021-09-25 00:41:29	14.9	0.9	5.8	16.9	48	0	1010.7	SE	13.9	49	18	0
2021-09-25 00:56:58	14.7	0.9	5.6	16.7	46	0	1010.6	SW	13.1	49	17.8	0
2021-09-25 01:12:28	14.4	0.9	5.6	16.6	44	0	1010.7	SW	13.6	49	17.7	0
2021-09-25 01:27:57	14.2	0.9	5.6	17	46	0	1010.9	S	15.0	49	17.6	0
2021-09-25 01:43:25	14.1	0.9	5.6	16.3	49	0	1010.8	W	14.8	49	17.5	0
2021-09-25 01:58:54	13.9	0.9	5.6	18.8	37	0	1010.8	SE	15.9	49	17.4	0
2021-09-25 02:14:23	13.8	0.9	5.6	16.3	47	0	1011	S	14.2	49	17.4	0
2021-09-25 02:29:52	13.6	0.9	5.6	16.4	45	0	1011	S	13.3	49	17.3	0
2021-09-25 02:45:19	13.5	0.9	5.6	16.3	48	0	1010.8	W	13.3	49	17.1	0
2021-09-25 03:00:50	13.3	0.9	5.6	16.3	46	0	1010.8	NE	12.4	49	16.9	0
2021-09-25 03:16:19	13.1	0.9	5.6	16	47	0	1010.8	S	13.7	49	16.8	0
2021-09-25 03:31:47	13	0.9	5.6	15.8	47	0	1010.7	S	12.9	49	16.7	0
2021-09-25 03:47:16	12.8	0.9	5.6	15.2	47	0	1010.9	S	12.0	49	16.6	0
2021-09-25 04:02:48	12.6	0.9	5.6	15.1	48	0	1010.8	S	12.7	49	16.4	0
2021-09-25 04:18:16	12.5	0.9	5.6	15.5	44	0	1010.8	S	10.9	49	16.3	0
2021-09-25 04:33:46	12.3	0.9	5.8	15.4	49	0	1011	S	11.7	49	16.2	0
2021-09-25 04:49:13	12.2	0.9	5.8	15.4	48	0	1011.1	S	13.0	49	16.1	0
2021-09-25 05:04:42	12.1	0.9	5.8	15.3	48	0	1011	S	13.7	49	16.1	0
2021-09-25 05:20:12	12	0.9	5.6	14.5	50	0	1011.2	S	13.7	49	15.9	2
2021-09-25 05:35:42	11.8	0.9	5.8	14.9	52	0	1011.2	S	13.4	49	15.8	3
2021-09-25 05:51:11	11.8	0.9	5.8	15.1	46	0	1011.3	S	14.6	49	15.9	15
2021-09-25 06:06:40	11.8	0.9	5.8	16.1	44	0	1011.4	S	14.5	49	15.9	21
2021-09-25 06:22:11	11.7	0.9	5.8	14.8	49	0	1011.5	S	14.5	49	15.8	59
2021-09-25 08:39:38	12.5	0.9	6.2	26.5	29	0	1011.2	S	12.6	49	17.5	349
2021-09-25 08:55:08	13	0.9	6.0	26.1	29	0	1011.5	SE	15.7	49	18.1	407
2021-09-25 09:10:37	13.7	0.9	6.0	26.8	29	0	1011.7	SE	13.3	49	18.8	458
2021-09-25 09:26:05	14.7	0.9	5.8	25.8	29	0	1011.8	E	21.3	49	19.4	510
2021-09-25 09:41:34	15.7	0.9	5.8	26.9	27	0	1011.9	NE	18.9	48	20	610
2021-09-25 09:57:02	16.4	0.9	5.8	28.1	26	0	1012	E	21.4	48	20.6	647
2021-09-25 10:12:31	17.4	0.9	6.0	28.6	25	0	1012.2	E	19.9	48	21.9	659
2021-09-25 10:27:59	18.8	0.9	5.8	29.3	27	0	1012.2	E	17.7	48	22.3	701
2021-09-25 10:43:28	19.9	0.9	5.6	30.1	24	0	1012.3	E	18.9	48	23.4	732
2021-09-25 10:58:58	20.2	0.9	5.8	30	25	0	1012.2	SW	23.2	48	25.2	779
2021-09-25 11:14:27	20.8	0.9	5.8	29.3	31	0	1012.5	SW	31.4	48	27.7	797
2021-09-25 11:29:56	21.5	0.9	6.0	29.1	32	0	1012.4	SE	33.7	48	28.4	820
2021-09-25 11:45:24	22.1	0.9	6.0	29.5	28	0	1012.2	SW	32.4	48	28.3	845

Detailed Bill of Materials and Comparative Cost Analysis of the Proposed Internet of Farming (IoF) Field Monitoring Station.

Table S3. Bill of Materials and Unit Cost of the Proposed IoF Field Monitoring Station

No	Component	Measured Parameter(s)	Unit Price ($)
1	ESP32-D0WD dual-core processor	Central processing / control	7.99
2	Raspberry Pi Zero Wireless	Auxiliary processing / high-speed interface	10.00
3	Air temperature & RH sensor	Air temperature; relative humidity	29.90
4	Barometric pressure sensor	Barometric pressure	10.90
5	Wind speed sensor	Wind speed (Hall effect)	45.00
6	Wind direction sensor	Wind direction (16-point)	45.00
7	Rainfall sensor	Rainfall (tipping-bucket, pulse)	29.90
8	Solar radiation sensor	Solar radiation (silicon-cell)	59.00
9	Leaf wetness sensor	Leaf wetness (0–15 scale)	195.00
10	Soil temp./moisture/EC probe	Soil temperature; VWC; electrical conductivity	99.00
11	Soil pH sensor	Soil pH	139.00
12	Cellular communication module	Data transmission to cloud (LTE CAT4, 150 Mbps downlink)	69.00
13	Imaging module	Field / canopy visual monitoring	≈25.00
14	Photovoltaic panel	Power supply (solar), 12 V/ 42 W monocrystalline solar panel	≈65.00
15	Battery	12 V / 7 Ah sealed lead-acid battery	≈22.00
		Subtotal, named components	851.69
		Enclosure, mounting mast, cabling, connectors, and RS485 bus interface hardware (not individually specified in the manuscript)	≈120–200
		Total per-unit field station cost	≈970–1,050

Table S4. Publicly Available Market Prices of Commercial Precision Agriculture Monitoring Systems.

Commercial Monitoring System	Scope of Measurement	Price ($)	Source
EnviroMonitor – My Farm, My Weather	Air temperature, relative humidity, wind speed, wind direction, rainfall, solar radiation, cloud connectivity	1,756	Davis Instruments
HOBO Weather Station Kit – Intermediate	Air temperature, relative humidity, wind speed, wind direction, solar radiation (optional), cloud-based environmental monitoring	2,363	Onset HOBO
HOBO RX3000 Remote Weather Station Starter Kit	Air temperature, relative humidity, wind speed, wind direction, cloud-based environmental monitoring	2,549	Onset HOBO
EnviroMonitor – IPM System	Weather monitoring, leaf wetness sensing, and integrated pest management (IPM) support	3,030	Davis Instruments
EnviroMonitor – Inversion System	Weather monitoring, temperature inversion, frost and spray drift monitoring	3,205	Davis Instruments
EnviroMonitor – Irrigation System	Weather monitoring, soil moisture sensing, and irrigation management	3,140	Davis Instruments
EnviroMonitor – Advanced Irrigation System	Weather monitoring, irrigation management, flow monitoring, soil moisture monitoring	3,735	Davis Instruments

Notes:

1. Prices were collected in July 2026 from official manufacturer websites and represent publicly available list prices at the time of data collection. Actual purchase prices may vary depending on system configuration, optional accessories, software subscriptions, shipping costs, regional distributors, and applicable taxes.
2. The commercial systems included in this comparison were limited to solutions with publicly available manufacturer list prices to ensure transparency and reproducibility of the cost analysis.
3. The per-unit cost of the proposed IoF field monitoring station is $970–1,050, including the enclosure, mounting mast, cabling, connectors, and RS485 interface hardware. In comparison, the commercial monitoring systems listed in Table S4 are priced from $1,756 to $3,735.

References:

1. Davis Instruments. *EnviroMonitor Agricultural Monitoring Systems.* Available online: https://www.davisinstruments.com/pages/enviromonitor (accessed on 27 July 2026).
2. Onset HOBO. *Weather Station Kits.* Available online: https://www.onsetcomp.com/weather-station-kits (accessed on 27 July 2026).
3. Onset HOBO. *HOBO RX3000 Remote Weather Station Starter Kit.* Available online: https://www.onsetcomp.com/products/kit/rx3004-sys-kit-813 (accessed on 27 July 2026).
